# Forecasts of climate-driven habitat suitability distribution are complicated by taxonomic and cytogenetic complexity in *Rhexia* (Melastomataceae)

**DOI:** 10.1093/aobpla/plag043

**Published:** 2026-09-23

**Authors:** Lyanna DeLeon, Karina Mendez, Jonathan Barz, Michelle L Gaynor, Makenzie E Mabry, Pamela S Soltis, Douglas E Soltis

**Affiliations:** Department of Life, Earth, and Environmental Sciences, West Texas A&M University, Canyon, TX 79016, United States; Biological Sciences, Chabot College, Hayward, CA 94545, United States; Florida Museum of Natural History, University of Florida, Gainesville, FL 32611, United States; Florida Museum of Natural History, University of Florida, Gainesville, FL 32611, United States; Department of Biology, University of Florida, Gainesville, FL 32611, United States; Ecology and Evolutionary Biology, University of Michigan, Ann Arbor, MI 48109, United States; Florida Museum of Natural History, University of Florida, Gainesville, FL 32611, United States; Florida Museum of Natural History, University of Florida, Gainesville, FL 32611, United States; Florida Museum of Natural History, University of Florida, Gainesville, FL 32611, United States; Department of Biology, University of Florida, Gainesville, FL 32611, United States

**Keywords:** climatic niche, ecological niche models, species distribution models, meadow beauty, polyploidy

## Abstract

*Rhexia* (Melastomataceae), a temperate genus composed of 13 species in eastern North America, provides a useful system for evaluating how taxonomic and cytogenetic complexity influence forecasts of climate-driven habitat suitability distribution. Ecological niche models incorporating climatic and edaphic variables were used to estimate current and future habitat suitability for diploid, polyploid, and mixed-cytotype species. Habitat suitability was strongly associated with soil texture, hydrological conditions, and precipitation, with suitable environments characterized by sandy, acidic, and seasonally moist habitats. Projections under future climate scenarios revealed highly variable, species-specific responses, with some taxa exhibiting substantial expansions in the distribution of suitable habitat and others experiencing pronounced contractions. Contrary to expectations, ploidal level was not consistently associated with niche breadth or climate response, as both diploid and polyploid species displayed a wide range of outcomes. These findings highlight the need for flexible, habitat-focused conservation strategies that consider hydrological and edaphic variables and demonstrate how taxonomic and cytogenetic complexity can complicate forecasts of biodiversity change under future climate scenarios.

## Teaser

Climate change is expected to alter the habitat suitability of plants. Ecological niche models can be used to predict these changes yet are made complicated by taxonomic and cytogenetic variation. *Rhexia* (Melastomataceae), a genus of 13 species in eastern North America, provides a useful system for examining these challenges. We evaluated how diploid, polyploid, and mixed-cytotype taxa may respond to future climate conditions and identified the environmental factors underlying differences in habitat suitability among species.

## Introduction

Predicting how species may potentially respond to climate change projections is a central challenge in ecology and conservation biology; such forecasts are increasingly used to inform biodiversity assessments and management strategies (IPCC 2021). Ecological niche models (ENMs) are widely used to project shifts in suitable habitat under projected future climate scenarios (Elith and Leathwick 2009, Peterson et al. 2011, Franklin 2023). However, the reliability of these projections depends on many assumptions including those involving species boundaries, assumptions that may be violated in taxonomically complex groups (Araújo and Peterson 2012, Guisan et al. 2017). Processes such as hybridization and polyploidy can blur species limits and increase ecological variation (Soltis and Soltis 2009). Together, these processes may generate dynamic and overlapping ecological niches, challenging the assumption that species represent discrete and stable units in ENM-based forecasts. As a result, ENM-based projections may misestimate species’ niche breadths, adaptive capacity, and ultimately their vulnerability or resilience to climate change. When taxonomic and cytogenetic complexities are unresolved, occurrence data may aggregate ecologically distinct lineages. This over-aggregation may lead to inflated niche breadth estimates, or it may mask environmentally specialized taxa. Despite increasing recognition of these issues, few studies have examined how variation in ploidy and hybridization influence ecological niche estimates and projections of future habitat suitability.

The angiosperm genus *Rhexia* (Melastomataceae), commonly known as meadow beauty, provides an ideal system in which to investigate the challenges reviewed above. Melastomataceae include few temperate species, with only two genera, *Rhexia* and *Miconia*, occurring in the United States (Renner 1993, Judd and Ionta 2022). Species of *Rhexia* are typically found in open, sunlit habitats such as wet meadows, pine flatwoods, savannas, and roadside margins, where they are associated with sandy, acidic, and poorly drained or seasonally saturated soils (Nesom 2012). The genus is distributed across eastern North America, spanning the Atlantic and Gulf Coastal Plains from Nova Scotia south to Florida and west to eastern Texas, with additional occurrences in the Caribbean, including Cuba, Hispaniola, and Puerto Rico (Nesom 2012). *Rhexia* species are generally early successional plants that occupy habitats maintained by disturbance, particularly fire and other perturbations (Kral and Bostick 1969, Godfrey and Wooten 1981, Wunderlin et al. 2017). Morphologically, the species of *Rhexia* are diverse, varying widely in floral structure, size, and colouration, including flowers that range from yellow and pale pink to deep purple or white (Fig. 1a). These species exhibit heteranthery and are primarily buzz-pollinated by bees capable of sonication, a pollination system that is characteristic of Melastomataceae (Renner 1989, Larson and Barrett 1999, Vallejo-Marín et al. 2010). In areas of sympatry, many species co-flower, facilitating interspecific pollen transfer and hybridization (Kral and Wurdack 1982, Ionta et al. 2007, Nesom 2012). Such gene flow may influence realized niche breadth and geographic distributions, complicating the interpretation of ENM-based projections. These dynamics are particularly relevant in regions of high co-occurrence, including central Florida and the southeastern USA (Fig. 1b).

**Figure 1:**
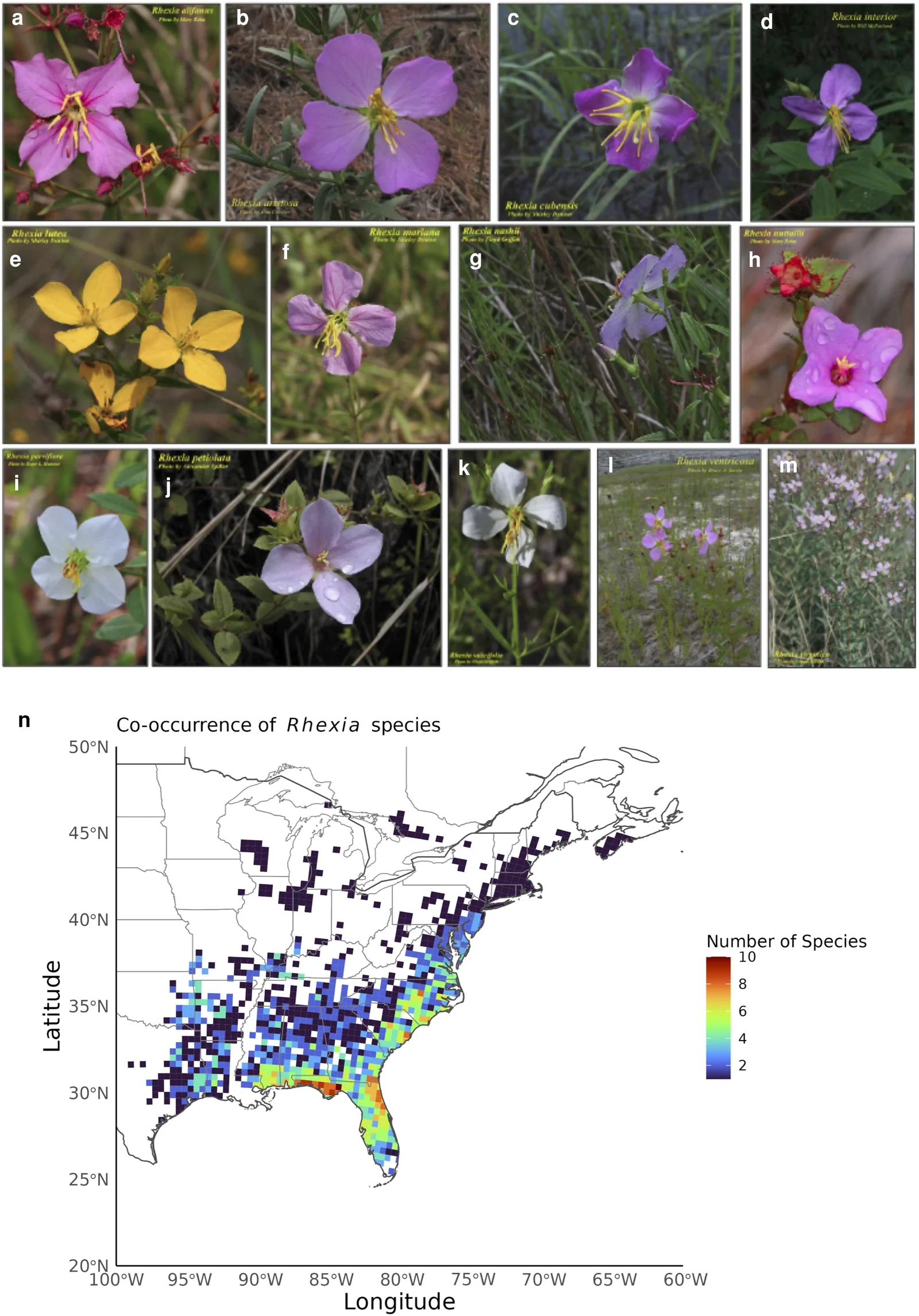
Photographs (from Atlas of Florida Plants; https://florida.plantatlas.usf.edu/) and distribution of *Rhexia* species analysed in this study: (a) *Rhexia alifanus* (Mary Keim), (b) *R. aristosa* (Alan Cressler), (c) *R. cubensis* (Shirley Denton), (d) *R. interior* (Will McFarland), (e) *R. lutea* (Shirley Denton), (f) *R. mariana* (Shirley Denton), (g) *R. nashii* (Floyd Griffith), (h) *R. nuttallii* (Mary Keim), (i) *R. parviflora* (Roger L. Hammer), (j) *R. petiolata* (Alexander Spiller), (k) *R. salicifolia* (Floyd Griffith), (l) *R. ventricosa* (Bruce A. Sorrie), and (m) *R. virginica* (Dennis Girard). (n) Map illustrating species co-occurrence across 40-km grid cells. Warmer colours (red, orange, and yellow) indicate areas where more *Rhexia* species co-occur, whereas cooler colours (blue and purple) indicate areas where fewer species co-occur. The map indicates a concentration of species in the southeastern coastal plain and Gulf region, with decreasing co-occurrence towards the northern and western extents.

Initially described by Linnaeus in 1753, the genus *Rhexia* underwent taxonomic scrutiny in the mid-20th century. James (1956) provided a foundational morphological classification, dividing 11 species into two series based on anther morphology: Series A, with straight, ascending anthers less than 2 mm long (*R. lutea*, *R. nuttallii*, and *R. petiolata*), and Series B, characterized by descending, curved anthers ranging from 4 to 11 mm (*R. alifanus*, *R. aristosa*, *R. cubensis*, *R. mariana*, *R. nashii*, *R. ventricosa*, and *R. virginica*). The placement of *R. parviflora* was less clear, as it possesses slightly curved anthers that do not fit neatly into either series. Advancements in molecular techniques allowed Ionta et al. (2007) to integrate DNA sequence data with 37 morphological characters, revealing three primary clades: the *R. petiolata* clade (*R. petiolata*, *R. nuttallii*, and *R. alifanus*), the *R. mariana* clade (*R. mariana*), and the *R. virginica* clade (*R. virginica* and *R. aristosa*). Notably, only 6 of the 11 species were included in this analysis, and the placements of the remaining species were not assessed. Building upon Ionta et al. (2007), Nesom (2012) proposed an infrageneric classification, recognizing 13 species and delineating four sections: Sect. *Rhexia*, encompassing species with unequal stem faces and longer anthers; Sect. *Cymborhexia*, characterized by *R. alifanus* with its unique morphological traits; Sect. *Brevianthera*, including species with shorter anthers and equal stem faces; and Sect. *Luteorhexia*, comprising *R. lutea*, distinguished by its yellow flowers.

Taxonomic treatments and species delimitation in *Rhexia* are further complicated by ploidal variation within and between species and known hybridization among species (James 1956, Kral and Bostick 1969, Ionta et al. 2007). Both may influence geographic distributions by facilitating gene flow across species and environmental gradients and by obscuring species boundaries. Seven species are exclusively diploid (*n* = 11; Fig. 3), while the remaining six species are either polyploids or contain multiple cytotypes (Kral and Bostick 1969, Ionta et al. 2007, Judd and Ionta 2022). The latter include *R. cubensis* (*n* = 11, 22, and 33), *R. virginica* (*n* = 11 and 22), and *R. nashii* (*n* = 22 and 33) (Judd and Ionta 2022). *Rhexia interior*, *R. lutea*, and *R. ventricosa* are tetraploid (*n* = 22). Notably, *Rhexia* chromosome numbers are often based on only a few or a single chromosome count per species. The origin and nature (allo- vs. autopolyploidy) of each polyploid lineage is mostly unknown; however, polyploid cytotypes in mixed-ploidy species are likely autopolyploids, although *R. nashii* may be an allopolyploid, with *R. mariana* and *R. virginica* its likely parents (Ionta et al. 2007). Detailed studies of cytotype distribution have not been conducted for species of *Rhexia*; however, the current ranges of the mixed-cytotype species suggest that ploidal variation does not consistently influence the distribution, as constricted and expanded ranges are observed in *R. cubensis* and *R. virginica*, respectively. *Rhexia cubensis*, with three cytotypes, is narrowly distributed, being found only in Florida and Cuba, and *R. virginica*, which occurs as a diploid and tetraploid, has a much wider distribution and occurs farther north (Canada) than any other species in Melastomataceae (Judd and Ionta 2022).

The southeastern USA supports a rich flora, much of which occurs within the North American Coastal Plain (NACP), an ecoregion extending from eastern Texas along the Gulf Coast and northward along the Atlantic seaboard to southern New Jersey. Recognized as a global biodiversity hotspot, the NACP harbours high levels of plant endemism despite its relatively low topographic complexity and environmentally homogeneous landscapes (Noss et al. 2015, Noss 2016). The biogeography of plant species in this region, including *Rhexia*, is strongly shaped by ecological factors such as edaphic conditions and disturbance regimes (White et al. 2026), particularly frequent, low-intensity fires that historically maintained open, sandy, herbaceous habitats. Disruption of these fire regimes through suppression and land-use change has promoted woody succession, reduced habitat availability, and altered community composition (Kong et al. 2021, White et al. 2022).

Together, the combination of taxonomic complexity, cytotype variation, and potential for gene flow among co-occurring species complicates efforts to define ecologically meaningful units within *Rhexia*. These challenges are further intensified by the relatively homogeneous environmental conditions of the NACP, where overlapping distributions and shared habitat preferences may obscure ecological differentiation among taxa. As a result, the extent to which projected climate change will alter the distribution and persistence of *Rhexia* species remains poorly understood.

Climate is now changing at an unprecedented rate (IPCC 2021), potentially outpacing many plant species’ ability to migrate or adapt (Corlett and Westcott 2013). As sessile organisms, plants have limited capacity to respond rapidly to changing conditions (Jump and Peñuelas 2005), and projected shifts in temperature, precipitation, sea level, and disturbance regimes are expected to alter habitat availability and population persistence across the NACP (Naranjo et al. 2022). Recent evidence already supports shifts in the distributions of numerous plant species, with many moving poleward and upward in elevation to follow suitable climates (Chen et al. 2011, Pecl et al. 2017, Lenoir et al. 2020), although the velocity of climate change may exceed the rate at which plants can track their optimal conditions (Loarie et al. 2009).

Given these interacting sources of biological and environmental complexity, *Rhexia* provides an ideal system for evaluating how taxonomic and cytogenetic variation influence ecological niche estimates and projections of climate-drivendistributional change of suitable habitat. To address this, we examine the potential impacts of projected climate change on abiotic niche suitability for *Rhexia* species using ecological and climatic niche modelling approaches. ENMs integrate species occurrence data with environmental variables to estimate habitat suitability based on ecological requirements, while climatic niche modelling emphasizes the characterization of climatic tolerances and the projection of geographic distributions under future climate scenarios (Wang et al. 2016). Specifically, we address two primary questions: (i) How will climate change potentially affect the distribution of suitable abiotic niches of *Rhexia* species? and (ii) Do species that are exclusively polyploid or contain mixed cytotypes exhibit broader niches than their diploid sister lineages?

## Methods

### Occurrence records and bioclimatic data processing

This study adopts a species concept informed by the most recent molecular and morphological insights (Judd and Ionta 2022) and consistent with Nesom’s (2012) taxonomic treatment, recognizing 13 species: *R. alifanus* Walt., *R. aristosa* Britton, *R. cubensis* Griseb., *R. interior* Pennell, *R. lutea* Walt., *R. mariana* L., *R. nashii* Small, *R. nuttallii* C. W. James, *R. parviflora* Chapman, *R. petiolata* Walt., *R. salicifolia* Karl & Bostick, *R. ventricosa* Fern. & Grisc., and *R. virginica* L. Global occurrence data for *Rhexia* species were retrieved using R v.4.2 from the Global Biodiversity Information Facility (GBIF) and Integrated Digitized Biocollections (iDigBio) on 29 November 2023, using the gatoRs package (Patten et al. 2024). Taxonomic resolution, including the identification of accepted synonyms, was based on the Atlas of Florida Plants (Wunderlin et al. 2025, https://florida.plantatlas.usf.edu/).

Occurrence records were filtered by removing any records without locality information or those that occurred in improbable locations (i.e. at longitude and latitude of 0). Latitude and longitude were then rounded to two decimals to match the precision of bioclimatic layers. The functions *cc_outl* and *cc_inst* from the package CoordinateCleaner v.3.0.1 were used to remove geographic outliers and records from botanical gardens, herbaria, universities, and museums (Zizka et al. 2019).

After removing duplicates (records with matching coordinates, year, month, and day), spatial thinning was utilized to reduce the effects of sampling bias and to retain one occurrence record per pixel (∼1 km^2^). Records were further reduced to be at least 5 km apart via the function *thin* in spThin v.1.7-29 to prevent over-fit models (Aiello-Lammens et al. 2015). Finally, occurrence records were visually inspected, noting the basis of the record (e.g. preserved specimen, living specimen, material sample, human observation, occurrence). Records that were human observations and extended the distribution of the species were removed as were any remaining erroneous records (e.g. in the ocean).

Environmental predictors were obtained from WorldClim v.2.1, comprising 19 bioclimatic and elevation variables at a resolution of 30 arc-seconds (∼1 km^2^) for the 1970–2000 time period (Fick and Hijmans 2017). Soil data were sourced from SoilGrids v.2.0 (Poggio et al. 2021) and resampled to match the resolution of the WorldClim layers. The spatial extent shared by all species was determined using the alpha hull approach implemented in the *getDynamicAlphaHull* function from the rangeBuilder package (Davis Rabosky et al. 2016), which created boundaries encompassing occurrence points. To approximate the accessible area (commonly referred to as M) for each species, a buffer zone was added to the hull based on the 80th quantile of the maximum distance between occurrence points, calculated using *st_distance* from the sf package (Pebesma 2018). This buffer was intended to reflect dispersal limitations and potential range dynamics of *Rhexia* species, which are typically associated with patchy wetland habitats and are not known to exhibit long-distance dispersal. Defining the calibration area in this way restricts model training to environments likely accessible to the species and reduces extrapolation into geographically or biologically unrealistic regions (Barve et al. 2011, Feng 2023). The environmental predictors were then cropped and masked to the buffered hull using the *crop* and *mask* functions from the raster package (Hijmans 2025), ensuring alignment between occurrence records and the environmental variables.

### Evaluating the realized niche of *Rhexia* species

For each occurrence record, the values of all environmental variables were extracted using the *extract* function from the raster package (Hijmans 2025). To analyse interspecific variation, a principal component analysis (PCA) was performed using the *prcomp* function in base stats (R Core Team 2024). The top-loading variables for each principal component (PC) were determined by calculating the relative contributions of variables to each axis, facilitating the interpretation of the most influential environmental factors separating species. To test for significant differences among species in the top-loading variables, one-way analyses of variance (ANOVAs) were conducted using the *aov* function (R Core Team 2024), followed by pairwise comparisons using Tukey’s honestly significant difference (HSD) test (*TukeyHSD*; Tukey 1949, R Core Team 2024). Significance groupings were summarized using compact letter displays implemented in the multcompView package (Graves et al. 2019).

### Development of ecological niche models

To minimize multicollinearity among predictor variables, variance inflation factors (VIFs) were calculated using the *vifstep* function from the usdm package (Naimi et al. 2014). VIF values exceeding a threshold of 10 were considered indicative of multicollinearity, and the corresponding variables were removed from the dataset. Of the original 81 environmental variables, 29 layers were selected for further analysis. These layers were saved in a new directory for subsequent modelling, ensuring a reduced risk of overfitting. Pearson’s correlation coefficients were also calculated between all environmental variables to identify potential relationships and redundancy among predictors. The *layerStats* function from the raster package (Hijmans 2025) was used to compute pairwise correlations, and the absolute values of these coefficients were saved as a correlation matrix. This matrix was used to evaluate and interpret the relationships between the top-contributing variables identified during subsequent ecological niche modelling.

ENMs were developed using the *ENMevaluate* function from the ENMeval package (Kass et al. 2021). Models were constructed with spatially thinned and cleaned species occurrence records and environmental layers filtered for multicollinearity using VIFs. A range of feature class combinations (e.g. L, LQ, LQH, and H) and regularization multiplier (rm) values (0.5–5) were tested to optimize model complexity. Background points (*n* = 10000) were randomly generated across the study area to adequately sample environmental conditions available to the species, and the Maxent algorithm (*maxent.jar*) was used to construct ENMs (Phillips et al. 2006, Phillips and Dudík 2008). Cross-validation was performed using block partitioning. Each block was iteratively used as a testing set while the remaining blocks served as the training data.

Model performance was evaluated using the Corrected Akaike Information Criterion (AICc), the average validation Area Under the Curve (AUC), and the average test omission rate, following guidelines from Radosavljevic and Anderson (2014) and Kass et al. (2021). The optimal model was selected based on the lowest AICc score, the lowest omission rate, and the highest AUC. Variable importance and response curves were generated for the selected models to identify key environmental predictors influencing the habitat suitability of each species.

### Current and future niche suitability projections

After constructing optimal ENMs for each species, we generated current projections to estimate the distribution of suitable habitat across the species’ accessible areas. These current habitat suitability maps serve as a baseline for assessing changes in the distribution of suitable habitat under future climate scenarios.

Future projections of habitat suitability were generated for two climate models (ACCESS-CM2; Bi et al. 2020, and GISS-E2-1-G; Kelley et al. 2020) across two future periods (2041–2060 and 2081–2100) and three shared socioeconomic pathways (SSPs 2–4.5, 3–7.0, and 5–8.5; O’Neill et al. 2017, Riahi et al. 2017). Future climate layers were obtained from WorldClim v.2.1 at a resolution of 30 arc-seconds (∼1 km^2^) and processed to ensure alignment with the current environmental layers. To maintain consistency in predictor variables, each climate model, SSP, and period was processed by stacking bioclimatic variables and then subsetting based on those identified in the VIF analysis. Edaphic (soil) and elevation variables were held constant across periods to isolate the impact of climate change on the distribution of habitat suitability. Using the optimal ENM for each species, these processed future layers were used to generate habitat suitability projections, providing insight into potential distributional shifts under various climate change scenarios.

To quantify inferred shifts in the distribution of suitable habitat over time, pairwise comparisons between current and future projections were conducted. Continuous habitat suitability maps were first transformed into binary classification maps, applying a threshold of ≥0.7 to categorize pixels as suitable or unsuitable. This threshold enabled consistent comparison of habitat suitability between periods and scenarios, identifying regions of habitat gain, loss, or stability. Using the biomod2 package (Guéguen et al. 2016), shifts in habitat suitability were calculated by comparing binary maps of current and future projections, with habitat changes categorized into four classes: lost, unchanged, not occupied, and occupied in the future. Resulting maps were overlaid with geographic boundaries and visualized using customized colour scales to distinguish areas of habitat loss, gain, and stability.

### Quantifying niche overlap across current and future climate projections

To assess the similarity of predicted habitat distributions among *Rhexia* species, we calculated niche overlap using Schoener’s *D* (Schoener 1968) and Warren’s *I* (Warren et al. 2008) statistics. Raster files of ENM projections were stacked and analysed for both current and future climate scenarios. The function *calc.niche.overlap* from the ENMeval package (Kass et al. 2021) was used to compute these metrics. Schoener’s *D* was used to measure absolute differences in habitat suitability across space between species, while Warren’s *I* emphasized areas of high probability of overlap. Because our use of ENMs assumes niche conservatism (i.e. that species’ fundamental niches remain constant), these metrics quantify how the geographic distributions of suitable habitat shift relative to one another across time, rather than changes in the niches themselves.

## Results

### Occurrence records and data cleaning

After data cleaning, the number of occurrence records for the 13 species of *Rhexia* was substantially reduced, reflecting the removal of duplicate, incomplete, or geographically implausible records to improve data quality and modelling accuracy (Supplementary Table S1). The cleaned dataset retained a total of 7654 records from an initial 16399 raw records. Occurrence records varied significantly by species, with *R. mariana* and *R. virginica* maintaining the highest number of records at 2930 and 1971, respectively, after cleaning. Conversely, species such as *R. parviflora* and *R. aristosa* had the lowest number of retained records, with only 9 and 25 records remaining, respectively. This variation highlights differences in data availability and geographic sampling effort among species. Notably, species with multiple cytotypes had a larger number of records: *R. cubensis* (*n* = 11, 22, 33; 247 records) and *R. nashii* (*n* = 22, 33; 433 records), compared to species with single cytotypes, such as tetraploids *R. ventricosa* (42 records) and *R. interior* (79 records). Unfortunately, cytotype information is rarely included in specimen metadata, limiting our ability to match ploidal levels to individual records. Cleaned occurrence records for all 13 *Rhexia* species across their geographic ranges highlight the concentration of most species in the southeastern USA, particularly within NACP (Supplementary Figure S1). We found that diploid *R. alifanus* exhibits a localized distribution in the southeastern USA, while widespread species such as diploid *R. mariana* and mixed-cytotype *R. virginica* (*n* = 11, 22) show broad geographic ranges. In contrast, narrowly endemic species, including diploids *R. aristosa*, *R. parviflora*, and *R. salicifolia*, have limited occurrence records, reflecting their restricted habitats. *Rhexia cubensis* displays a unique range extending into the Caribbean region.

### The realized niche space

Initially, the PCA revealed multiple unusual outliers belonging to *R. cubensis*; these occurrence records were traced back to georeferenced records from Palm Beach County. These records, assigned to the county centroid, were found to overlap with a stormwater treatment facility, introducing distortion in environmental variables, particularly nitrogen levels, which were abnormally elevated. To address this issue, all occurrences georeferenced to any county centroid were excluded from further analyses.

After removing records georeferenced to the county centroid, the PCA revealed clustering patterns across the first four principal components, with PC1 explaining 26.4% of the variance and PC2 contributing 22.6%, while PC3 and PC4 explained 11.9% and 8.6%, respectively (Fig. 2a and b). The PCA revealed that *R. virginica*, a mixed-cytotype species, occupies some distinct niche space, likely attributable to the cooler temperatures and other environmental conditions associated with its occurrence at higher latitudes. To further examine species differences in the environmental variables contributing most strongly to the PCA, one-way ANOVAs followed by Tukey’s HSD *post hoc* tests were conducted on the two highest-loading variables for PC1, isothermality (BIO3) and soil carbonyl sulphide concentrations at 0–30 cm, and the highest-loading variable for PC2, nitrogen concentration at 0–5 cm (Fig. 2c–e). All three variables differed significantly among species according to one-way ANOVAs (isothermality: *F*_12_,_7018_ = 374.9, *P* < .001; soil carbonyl sulphide: *F*_12_,_7018_ = 110.6, *P* < .001; nitrogen: *F*_12_,_7018_ = 120.0, *P* < .001). Tukey’s HSD *post hoc* tests indicated that isothermality, which quantifies daily temperature oscillations relative to annual temperature oscillations, was significantly lower for *R. virginica* than for all other species (adjusted *P* < .01 for all pairwise comparisons). Soil carbonyl sulphide concentrations were lower for *R. salicifolia* than for 11 of the 12 other species (adjusted *P* < .05), whereas nitrogen concentrations were higher for *R. nuttallii* than for 11 of the 12 other species (adjusted *P* < .01); in both cases, the only nonsignificant comparison was with *R. parviflora*.

**Figure 2:**
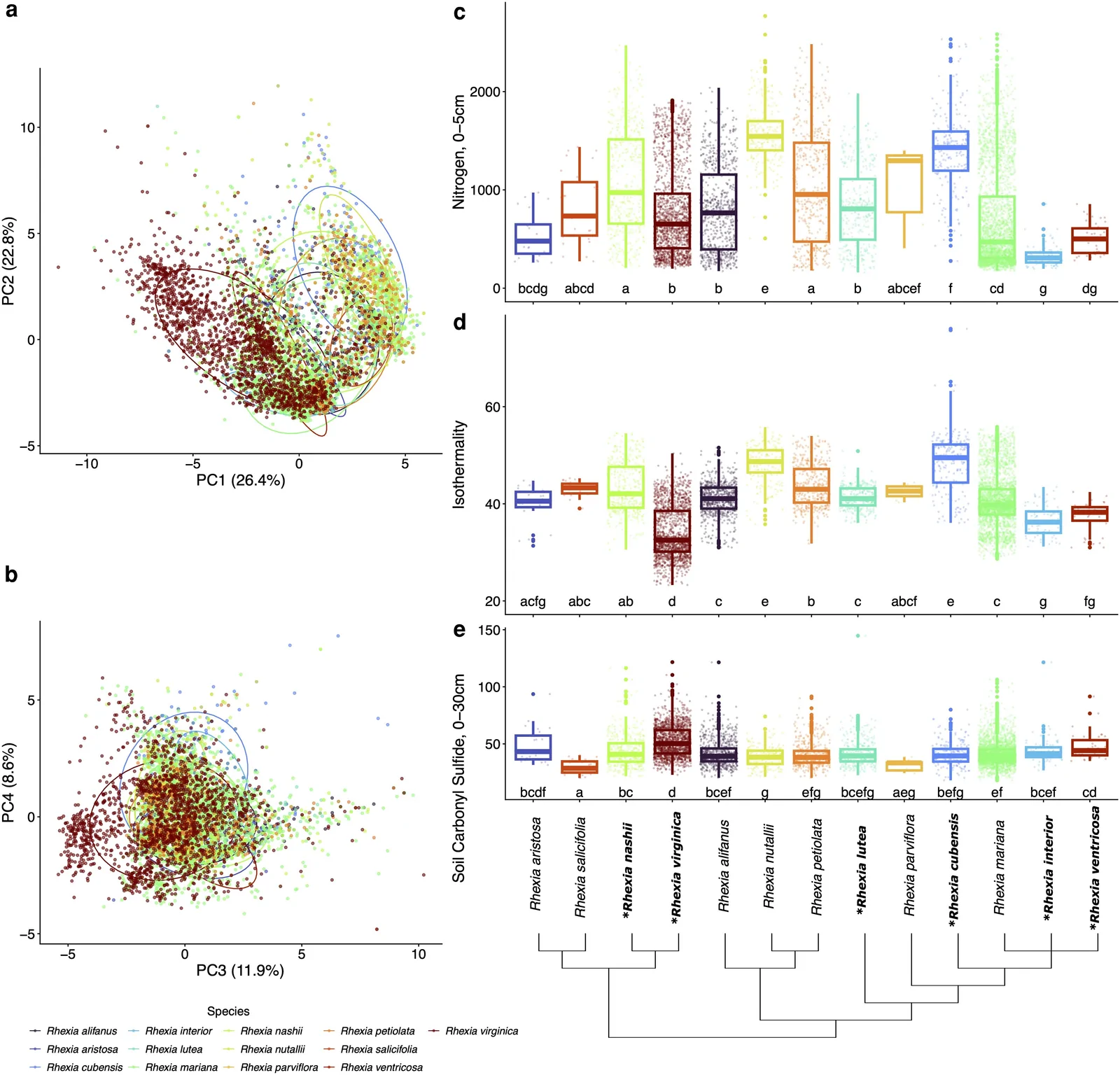
Principal component analysis (PCA) and box plot analyses of the environmental niches of *Rhexia* species. (a, b) PCA plots illustrate the distribution of 13 *Rhexia* species across the first four principal components (PC1, PC2, PC3, and PC4), which together account for 69.7% of the total variation (26.4%, 22.8%, 11.9%, and 8.6%, respectively). Ellipses represent 95% confidence intervals for each species. (c-e) Box plots display variation in the top-loading variables from PC1 and PC2, nitrogen levels (0–5 cm depth), isothermality (BIO3), and soil carbonyl sulphide levels (0–30 cm depth). Letters beneath the box plots indicate significant differences between species based on Tukey’s HSD test. (Bottom right) A phylogeny (based on Michelangeli et al. 2013) provides a best estimate of species relationships, with polyploid species indicated in bold and mixed-cytotype species in bold with an asterisk (*).

### Assessment of niche models and contributions of bioclimatic variables

When assessing the optimal models selected for *Rhexia* species, a linear feature class with a regularization multiplier of 4.5 was the most frequently chosen model, appearing four times, followed by mixed linear and quadratic feature class with a regularization multiplier of 4.5, which was selected three times (Supplementary Table S2).

When combining the frequencies of top variables and their correlated counterparts, some patterns emerge in the environmental factors influencing *Rhexia* habitat suitability (Supplementary Table S2). Silt content at 100–200 cm depth was the most frequently selected variable, appearing eight times and exhibiting strong correlations with sand and silt across all depth intervals, underscoring the importance of soil type in habitat suitability. Volumetric fraction of coarse fragments (cfvo) also contributed prominently, appearing six times and showing consistent correlation with elevation, suggesting an association between substrate coarseness and topographic position. Additionally, precipitation-related bioclimatic variables—precipitation of warmest quarter (BIO18), precipitation of wettest month (BIO13), and precipitation of wettest quarter (BIO16)—each appeared six times, reinforcing the critical role of rainfall patterns in defining species niches.

Response curves (Supplementary Figure S2) illustrate how the top-contributing environmental variables influence species’ habitat suitability probabilities. While responses vary among species, many exhibited decreasing suitability with increasing silt content, particularly at depth, suggesting that habitats dominated by fine silt particles (≥0.002 mm and ≤0.05/0.063 mm) may be less suitable for some taxa. Coarse fragment volume showed a consistent negative relationship with suitability across all models, likely reflecting reduced soil water retention and rooting potential in rockier substrates. A similar pattern was observed for elevation, where lower suitability at higher elevations further supports *Rhexia*’s association with finer soils and low-lying habitats.

Conversely, precipitation-related bioclimatic variables, particularly precipitation of the warmest quarter (BIO18), along with the wettest month (BIO13) and driest month (BIO14), show predominantly increasing or flat responses, suggesting that *Rhexia* species favour wetter or seasonally moist habitats, with suitability increasing alongside rainfall up to a threshold. Temperature-related variables such as mean temperature of the wettest quarter (BIO8) and driest quarter (BIO9) exhibit more variable responses, indicating possible species-specific temperature tolerances or indirect climatic effects. Additionally, increasing soil pH consistently decreased suitability across models, suggesting a preference for acidic soils, while nitrogen availability showed mixed responses, potentially reflecting adaptation to low-nutrient environments. These combined patterns indicate that *Rhexia* habitat suitability is shaped by the interplay of hydrological regime, soil physical and chemical properties, and species-specific responses to climate and edaphic constraints.

When these models are projected onto current environmental conditions (Supplementary Figure S3), they revealed how niche models translate into geographic space. Species such as diploid *R. mariana* and mixed-cytotype *R. virginica* exhibit broad predicted distributions, suggesting greater ecological tolerance to a range of environmental conditions. In contrast, species such as diploids *R. parviflora* and *R. salicifolia* have more restricted ranges, reflecting narrower environmental preferences.

### Future projections of habitat suitability

ENMs for *Rhexia* species were projected under two future climate models (ACCESS-CM2 and GISS-E2-1-G) for the mid- (2041–2060) and late-century (2081–2100) periods across three SSPs (2–4.5, 3–7.0, and 5–8.5). Species exhibited varying trends in climatic suitability, with some experiencing substantial potential range contractions and others showing potential expansions (Fig. 3; Supplementary Figures S4–S15). Under the ACCESS-CM2 model, changes in climatic suitability varied widely among species. Several species experienced notable declines in suitable area. Tetraploid *R. ventricosa* displayed the most pronounced reductions, with suitability values decreasing by 66%–75% across mid-century scenarios and further reductions under late-century projections (Figs. 3 and 4a–c). Under the ACCESS-CM2 SSP5-8.5 late-century projection (2081–2100), *R. ventricosa* showed extensive losses of suitable area with relatively few newly suitable areas, resulting in a net contraction of climatically suitable area. Mixed-cytotype *R. cubensis* also showed consistent losses in suitable area, particularly under SSP3-7.0 and SSP5-8.5, with values decreasing by more than 40% across projections. Conversely, tetraploid *R. interior* and *R. lutea*, as well as diploid *R. parviflora*, showed notable increases in suitable area, particularly under SSP2-4.5 and SSP5-8.5. *Rhexia parviflora* showed an expansion under the late-century SSP5-8.5 projection, particularly across the southeastern USA (Fig. 4d–f). Under the GISS-E2-1-G model, species exhibited similar but sometimes more pronounced trends. Tetraploid *R. interior* and diploid *R. parviflora* again demonstrated significant gains in suitable area, particularly under SSP5-8.5 by 2081–2100. However, tetraploid *R. ventricosa* and mixed-cytotype *R. cubensis* experienced severe losses in suitable area, with *R. ventricosa* showing reductions exceeding 80% under SSP5-8.5.

**Figure 3:**
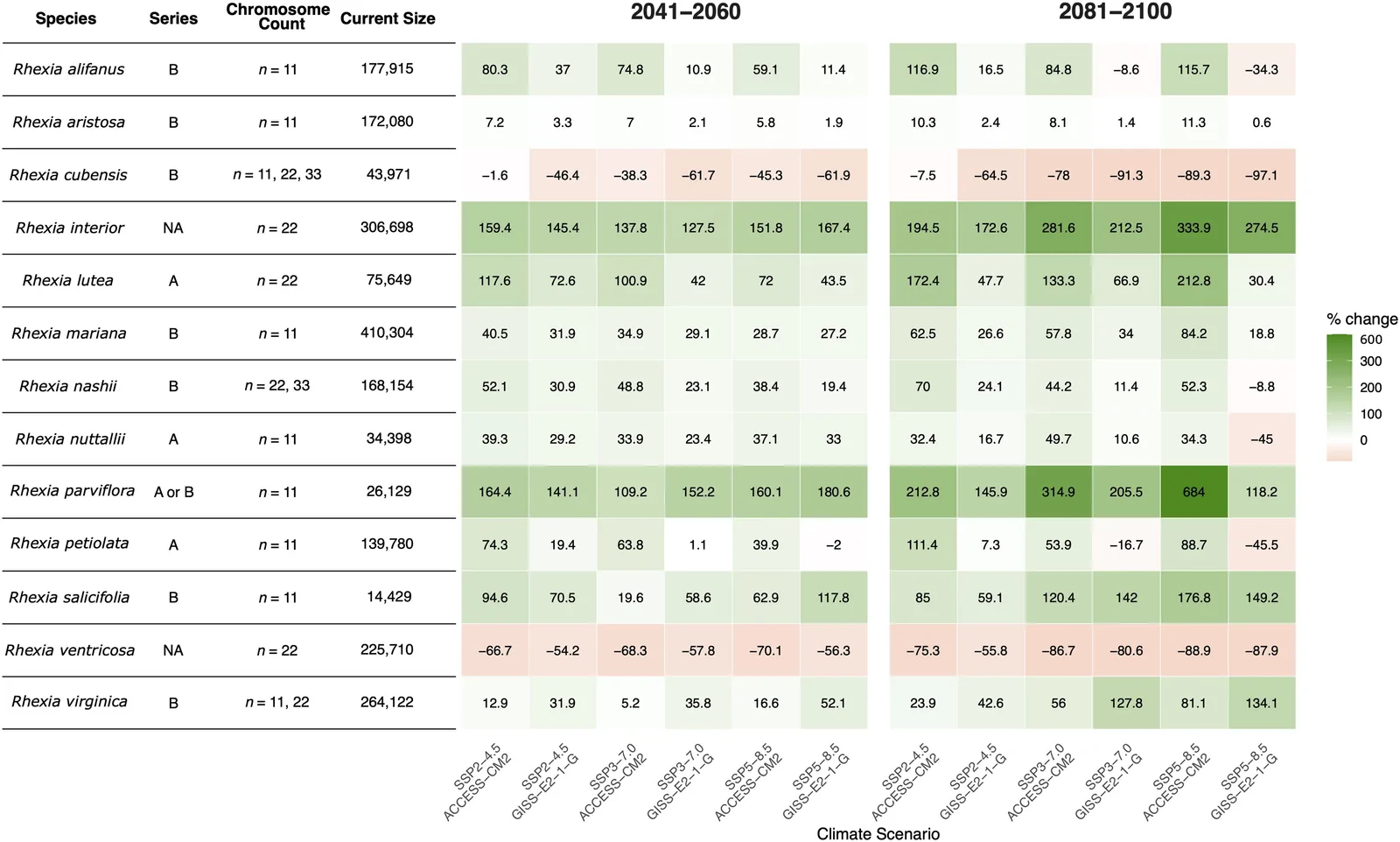
Current and projected changes in suitable habitat for *Rhexia* species under future climate scenarios. The table summarizes each species, taxonomic series, chromosome counts, and current number of suitable pixels (∼1 km^2^ each). The heatmap shows the projected per cent change in suitable habitat relative to current conditions under two climate models (ACCESS-CM2 and GISS-E2-1-G), two projection periods (mid-century; 2041–2060 and late-century; 2081–2100), and three shared socioeconomic pathways (SSP2-4.5, SSP3-7.0, and SSP5-8.5). Green indicates an increase and red indicates a decrease in projected suitable habitat.

**Figure 4:**
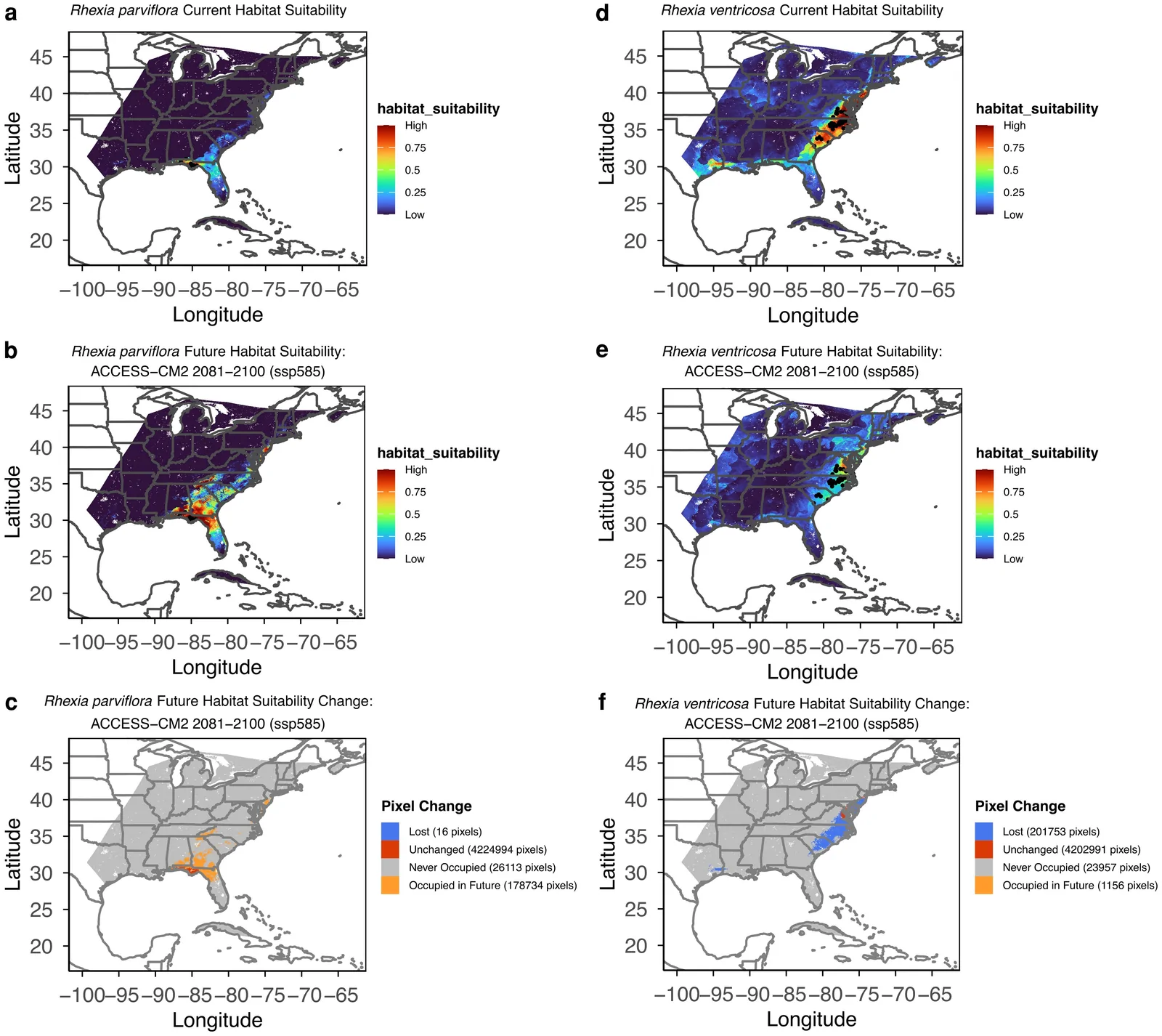
Habitat suitability and projected changes for tetraploid *Rhexia ventricosa* and diploid *R. parviflora* under current and future climate conditions (ACCESS-CM2, SSP5-8.5, 2081–2100). Current habitat suitability for (a) *R. ventricosa* and (d) *R. parviflora*, with a gradient from low (blue) to high (red) suitability. Future habitat suitability projections under the ACCESS-CM2 climate model, SSP5-8.5 scenario, for (b) *R. ventricosa* and (e) *R. parviflora*, with the same gradient. Changes in habitat suitability for (c) *R. ventricosa* and (f) *R. parviflora*, categorized as lost (blue), unchanged (grey), not occupied (white), and newly occupied in the future (orange) with noted pixel counts (∼1 km^2^ per pixel) for each category.

However, there were notable discrepancies between the ACCESS-CM2 and GISS-E2-1-G models, particularly under late-century (2081–2100) projections for SSP3-7.0 and SSP5-8.5. While diploids *R. alifanus*, *R. nuttallii*, and *R. petiolata* exhibited considerable increases in the amount of suitable habitat under ACCESS-CM2, these same species experienced significant habitat loss under GISS-E2-1-G. For instance, *R. petiolata* expanded substantially under ACCESS-CM2 and SSP5-8.5, but under GISS-E2-1-G, the extent of suitable habitat declined in the same scenario. These differences among climate projections highlight the uncertainty in species-specific responses and the contrasting trajectories that may occur under future climate conditions.

### Variation in niche similarity among *Rhexia* species

Although for polyploid *R. nashii* the likely parental species are *R. mariana* and *R. virginica*, the analysis of niche overlap revealed that diploid *R. petiolata* and *R. nashii* currently exhibit the highest ecological similarity in both Schoener’s *D* and Warren’s *I* metrics, with values of *D* = 0.87 and *I* = 0.98 (Fig. 5). This indicates that areas with predicted niche suitability are highly overlapping in both absolute and probability-weighted measures. Conversely, *R. parviflora* and *R. interior* showed the lowest niche similarity, with *D* = 0.03 and *I* = 0.13, suggesting minimal overlap between their suitable niche space.

**Figure 5:**
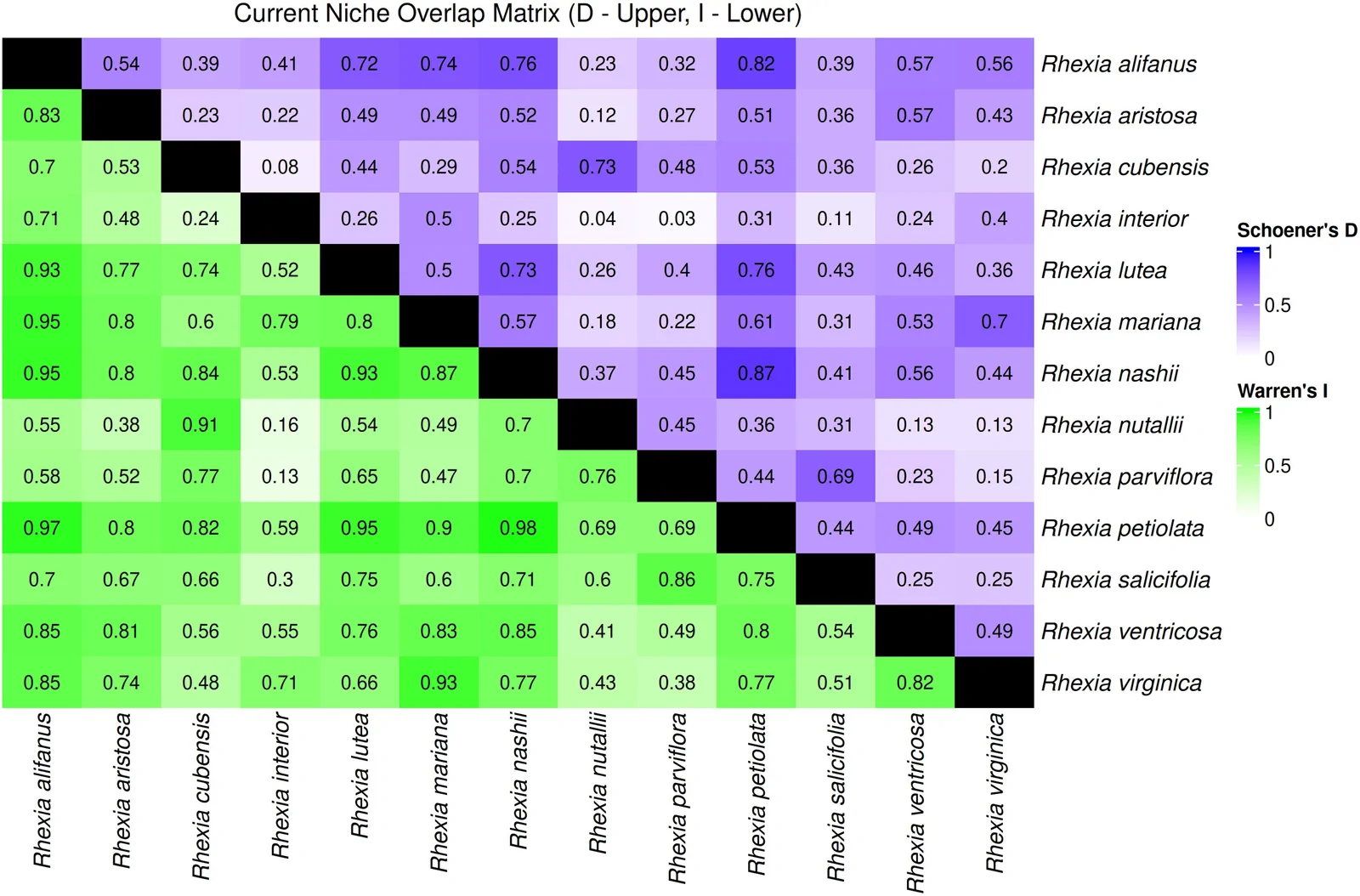
Climatic suitability overlap matrix for *Rhexia* species under current climatic conditions. Schoener’s *D* values (upper triangle, purple) represent absolute overlap in climatic suitability, while Warren’s *I* values (lower triangle, green) represent probability-weighted similarity in climatic suitability. Higher values indicate greater similarity in predicted climatic suitability between species.

Overall, the overlap among *Rhexia* species remains relatively stable across future climate models, with only minor fluctuations in similarity (Supplementary Figure S16). A few species pairs, such as *R. alifanus* and *R. parviflora* and *R. interior* and *R. salicifolia*, show slight increases in overlap, suggesting that their climatically suitable habitats may become more geographically aligned. Conversely, pairs such as *R. alifanus* and *R. nashii* and *R. lutea* and *R. nashii* show small decreases in overlap, indicating that their suitable habitats may become more spatially distinct under future climate scenarios. Because the underlying niches are assumed to be conserved, these patterns reflect changes in the geographic distribution of suitable habitat rather than changes in the niches themselves.

## Discussion

Understanding how taxonomic uncertainty, cytogenetic complexity, and frequent sympatry influence ecological niche projections is critical for predicting species responses to climate change. A central goal of this study was to evaluate whether variation in ploidy contributes to differences in ecological niche breadth and projected climate responses in *Rhexia*. Our results highlight several key patterns. First, data cleaning and validation substantially influenced model inputs, demonstrating how georeferencing uncertainty and inaccurate occurrence records can bias environmental estimates and niche characterization. Second, species of *Rhexia* exhibit highly variable responses to projected climate change, with some showing substantial expansions in suitable habitat and others experiencing pronounced contractions. This varied response is noteworthy given the frequent overlap in the current distributions of some species. Third, ploidal level is not consistently associated with niche breadth or climate response, as both diploid and polyploid taxa exhibit a wide range of potential outcomes. However, interpretations should be made cautiously given that information on ploidy remains sparse for many of these species. Together, these findings emphasize that accurate prediction of species responses to climate change depend on the quality of the underlying occurrence data and the biological complexity of the system, highlighting the challenges of forecasting species responses in taxonomically and cytogenetically variable groups.

### Occurrence validity and implications

Georeferencing accuracy is a critical factor in ENMs, as imprecise location data can introduce significant uncertainty into estimates of environmental conditions and predicted species distributions. Previous work has demonstrated that coordinate uncertainty in digitized natural history collection records can propagate into species distribution models and influence estimates of potential distributions (Marcer et al. 2022). In this study, data cleaning and validation substantially influenced model inputs, demonstrating how georeferencing uncertainty can distort environmental estimates and bias niche characterization. During our review of occurrence records, we identified samples that were georeferenced using county centroids, including three GBIF records (key = 1990258105, 122997717, and 4153121224) and one iDigBio record (uuid = 7ab00b07-9662-48bc-bd0e-a674b1d97099). These records overlapped with a stormwater treatment facility, resulting in artificially elevated nitrogen values and distorted environmental conditions.

Further investigation revealed that many older records had been assigned to county centroids, a common practice that can misrepresent the environmental conditions where species actually occur. This issue has been well documented in biogeographic studies, where centroid-based assignments can lead to erroneous ecological interpretations (Park and Davis 2017, Marcer et al. 2022). These results underscore the importance of verifying georeferenced data before incorporating them into ecological models. Future studies should prioritize high-precision locality data and, as we did here, apply correction methods or exclude centroid-based occurrences to improve the accuracy of niche modelling efforts.

### Species-specific responses to climate change

Projected changes in habitat suitability indicate that *Rhexia* species will each respond differently to future climate conditions (Chen et al. 2011, Pecl et al. 2017, Lenoir et al. 2020). Some species, such as *R. interior* and *R. parviflora*, consistently show increases in suitable habitat across scenarios, whereas others, including *R. ventricosa* and *R. cubensis*, exhibit substantial declines. These contrasting responses suggest that species-specific ecological tolerances and geographic constraints play a dominant role in shaping climate responses. However, the interpretation of these patterns may depend, in part, on taxonomic treatment. For example, *R. interior* has previously been treated as a variety of *R. mariana* (Kral and Bostick 1969), and combining or separating such taxa could alter estimates of niche breadth and projected climate responses. In this study, *R. interior* and *R. mariana* exhibited distinct responses to climate projections, suggesting that modelling these taxa separately captures ecological differences that would be obscured under a more inclusive taxonomic treatment. More broadly, this highlights that the taxonomic scale at which ENMs are constructed (e.g. species vs. infraspecific units) can influence both the magnitude and interpretation of predicted range shifts, as alternative taxonomic treatments may capture or obscure ecologically meaningful variation (Peterson and Navarro-Sigüenza 1999, Guisan et al. 2017). Differences between the projections for each species under each climate model further highlight uncertainty in the magnitude and direction of change, particularly under late-century scenarios (Araújo and New 2007).

High values of Schoener’s *D* and Warren’s *I* indicate that some *Rhexia* species share similar patterns of predicted climatic suitability under current climatic conditions. In other words, the environmental conditions identified by the models as suitable for these species are often similar, suggesting comparable climatic associations. However, high overlap does not necessarily imply that species occupy identical ecological niches, interact directly, or experience the same ecological pressures. Instead, it may reflect the resolution and scope of the environmental predictors included in the models, which may not capture important sources of ecological differentiation such as fine-scale habitat characteristics, edaphic variation, or biotic interactions. Notably, despite these similarities in current climatic suitability, projected responses to future climate change varied among species, indicating that shared climatic associations under present conditions do not necessarily translate into similar future distributional trajectories. From a biogeographic perspective, these results suggest that future distributions across the NACP will be restructured through individual species-specific responses rather than uniform range shifts. This pattern is consistent with previous work on Coastal Plain endemics, which found substantial heterogeneity in projected climate responses among closely related scrub-adapted taxa, with some species experiencing severe habitat loss while others showed more limited changes (Naranjo et al. 2022).

### Ploidy and ecological niche differentiation

Although polyploidy has been associated with increased ecological tolerance and diversification, we found no consistent relationship between ploidy and niche breadth or projected climate response across *Rhexia* species. Both diploid and polyploid species displayed a wide range of outcomes, including expansions and contractions in suitable habitat. Mixed-cytotype species such as *R. nashii* (*n* = 22, 33) showed moderate increases in predicted habitat suitability under ACCESS-CM2 but more uncertain responses under GISS-E2-1-G. In contrast, *R. cubensis* (*n* = 11, 22, 33) is projected to experience substantial habitat suitability loss across both models. Similarly, diploid species (*n* = 11) exhibited highly variable responses, with some (e.g. *R. alifanus*, *R. petiolata*) showing expansion under ACCESS-CM2 but declines under GISS-E2-1-G.

However, projections for *R. ventricosa*, a tetraploid species, show consistent habitat suitability loss across all scenarios, with stable distributional overlap suggesting contraction rather than expansion into new areas. These interpretations are constrained by the limited availability of cytogenetic data, as most specimens used in species distribution modelling lack cytotype information and published ploidy estimates are frequently derived from a small number of sampled individuals. Consequently, the extent of within-species cytotype variation and its influence on climatic responses remain poorly understood.

Research on niche differentiation in polyploids suggests that genetic factors, such as fixed heterozygosity and increased genomic diversity, may enhance their ability to tolerate a range of abiotic conditions (McIntyre 2012, Van De Peer et al. 2021). However, ecological differentiation among cytotypes is not universal, and niche overlap between polyploids and their progenitors is common (Glennon et al. 2014, Marchant et al. 2016, Parisod and Broennimann 2016, Mata et al. 2023). For example, in *Claytonia tennesseensis*, an allopolyploid, significant niche overlap exists with its diploid progenitors, *C. protrusa* and *C. bulbifera* (McIntyre 2012). Similarly, different ploidal levels of *Galax urceolata* often occupy overlapping climatic niches rather than distinct ones (Gaynor et al. 2018). Conversely, Muñoz-Pajares et al. (2018) demonstrated that diploid and tetraploid cytotypes of *Erysimum mediohispanicum* occupied subtly but significantly different ecological niches, suggesting that polyploidization can also contribute to ecological differentiation among cytotypes. While some polyploids, such as *Polystichum saximontanum*, have diverged significantly from their progenitors and adapted to novel ecological conditions, others remain associated with similar environments to their diploid parents (Marchant et al. 2016). For mixed-cytotype *Rhexia* species, very little is known about each cytotype’s distribution or gene flow among cytotypes; additional studies are needed to identify the likely ecological relationship among polyploids and their diploid progenitors for these species.

Our results are consistent with this broader pattern, indicating that cytogenetic complexity alone does not reliably predict ecological differentiation or responses to climate change. Although ploidal level may influence species responses, the direction and magnitude of these effects appear to be highly species-specific (Meirmans and Kolář 2025). Future studies incorporating cytotype-confirmed occurrences, detailed abiotic and biotic variables, genomic data, and broader phylogenetic context will be necessary to determine how cytotype variation influences ecological adaptation within *Rhexia*.

### Conservation implications for *Rhexia*

Habitat suitability in *Rhexia* is strongly shaped by soil texture, hydrological conditions, and precipitation patterns. Suitable environments are associated with sandy, low-silt substrates, acidic soils, and low-lying environments, while increasing coarse fragments and elevation reduce suitability. Precipitation variables indicate that many species depend on seasonally moist or hydrologically stable conditions. These patterns are consistent with the known ecology of *Rhexia*, which is typically associated with sandy, acidic, and poorly drained wetland habitats (Nesom 2012). Together, these features define the key environmental conditions that should be prioritized for habitat conservation.

The wetland environments inhabited by *Rhexia* support diverse plant communities, including *Sarracenia purpurea* (pitcher plants), *Eriocaulon compressum* (flattened pipewort), *Drosera capillaris* (pink sundew), and *Xyris* spp. (yellow-eyed grasses), and represent some of the most biodiverse yet vulnerable ecosystems in the NACP. However, these systems are increasingly threatened by land-use changes, hydrological alteration, and climate-driven shifts in precipitation (Mitsch and Gosselink 2015). Across the NACP, landscape-scale wetland degradation has been documented, including a loss of ∼13682 km^2^ of coastal forested wetlands between 1996 and 2016 due to intentional drainage, increased agricultural use, and ongoing climate-related pressures (White et al. 2022). Because *Rhexia* species are strongly tied to soil and moisture conditions, even modest changes to drainage, water availability, or substrate composition may substantially reduce habitat suitability. Although our models focus on climatic predictors, integrating land-use change and hydrological modification into future modelling efforts will be critical for more accurately forecasting species distributions.

Biological processes may further mediate species responses to environmental change. *Rhexia* species exhibit heteranthery, with buzz-pollinated flowers (Larson and Barrett 1999) and frequently co-flower in sympatry, creating opportunities for interspecific pollen transfer and reproductive interactions (Dellinger et al. 2025). Reproductive success may therefore depend on pollinator availability and behaviour. In *R. virginica*, pollen limitation associated with variable pollinator visitation has been documented (Larson and Barrett 1999), indicating that changes in pollen delivery can influence reproductive output. However, recent work on *R. mariana* found weak pollinator specialization and no effect of congeneric co-flowering context on visitation rates or pollination performance, suggesting that co-flowering may have neutral effects on pollination (Dellinger et al. 2025). Although direct evidence linking climate change to changes in pollinator interactions in *Rhexia* is currently lacking, climate-driven shifts in plant–pollinator phenology have been documented across multiple systems and may represent an additional vulnerability for pollinator-dependent species (Forrest 2015). Many wetland and seep habitats occupied by *Rhexia* species have experienced historical alteration and fragmentation throughout the southeastern USA, which may constrain population connectivity and limit the ability of species to track shifting climatic conditions (White et al. 2022). Together, reproductive interactions, habitat connectivity, and other biotic processes represent important factors that may contribute to variation in *Rhexia* responses to environmental change.

Consistent with these interacting factors, climate projections indicate that *Rhexia* species will respond heterogeneously to future environmental change. Differences among climate models highlight considerable uncertainty in both the magnitude and direction of projected range shifts. From a conservation perspective, this variability underscores the need for flexible, scenario-based strategies that account for multiple possible futures. Species projected to experience consistent habitat loss across models may warrant prioritization for conservation actions, including habitat protection, restoration, or ex situ strategies such as seed banking (Heller and Zavaleta 2009, Lawler 2009, Schwartz et al. 2012). In contrast, species projected to expand their ranges may still be limited by dispersal barriers, fragmentation, or biotic constraints, meaning that projected gains in suitable habitat may not translate into realized range expansion. The conservation status of *Rhexia* species already reflects substantial variation in vulnerability, likely linked to ecological specificity and exposure to habitat degradation. For instance, *R. virginica* is classified as least concern by the IUCN (Maiz-Tome 2016), while *R. mariana* is considered globally secure (G5) by NatureServe but listed as Endangered in Massachusetts. *Rhexia aristosa*, ranked as vulnerable (G3), is more restricted in distribution and sensitive to habitat loss, a pattern shared by other narrow endemics such as *R. salicifolia* (G3) and *R. parviflora* (G2: imperilled) (NatureServe 2024). Although formal conservation assessments are lacking for several species, regional variation in risk suggests that habitat specificity and anthropogenic pressures are key determinants of vulnerability.

Future research should focus on improving predictions of species persistence by integrating species-specific ecological constraints, dispersal capacity, and habitat connectivity. Resolving the distributions and ecological differentiation of cytotypes will be particularly important for understanding variation within the genus. In addition, incorporating paleoclimate reconstructions (e.g. last interglacial or last glacial maximum conditions) may provide insight into how historical climate dynamics have shaped diversification and endemism in the NACP, a region characterized by complex climatic and geological history (Noss et al. 2015, White et al. 2026). Together, these approaches will improve the ability to link ENMs with evolutionary and ecological processes, ultimately strengthening conservation strategies for *Rhexia* and the wetland ecosystems they inhabit.

## Conclusion

Building reliable ENMs for *Rhexia* species presents significant challenges due to shifting taxonomy and limited data on chromosome number within and across species. Taxonomic revisions and uncertainties in species delimitation complicate dataset consistency, making it difficult to accurately model species distributions. Additionally, because ploidal levels are often undocumented or inaccessible, models inevitably generalize across multiple cytotypes, potentially obscuring ecological and evolutionary differences between diploids and polyploids. As a result, current models represent an amalgamation of environmental responses across ploidal levels, rather than capturing the potentially unique niche characteristics of each cytotype. Addressing these challenges will require greater taxonomic resolution and expanded cytogenetic sampling. Future efforts could integrate chromosome counts from newly collected populations, genome size estimates obtained through flow cytometry, and emerging genomic approaches for estimating ploidy from sequence data (Gaynor et al. 2024). Linking these cytotype-confirmed datasets with georeferenced occurrence records would enable more precise ecological predictions and provide new opportunities to evaluate how polyploidy influences species distributions and adaptation in *Rhexia*.

## Supplementary Material

plag043_Supplementary_Data

## Data Availability

All scripts used in this study are available at GitHub (https://github.com/mmabry/Rhexia_ENMs). Raw and cleaned datasets, as well as current and future projections as tif files, are archived and accessible via Zenodo (10.5281/zenodo.15838288).
