# Supplementary material for: Forecasts of climate-driven habitat suitability distribution are complicated by taxonomic and cytogenetic complexity in Rhexia (Melastomataceae)
Source: AoB Plants. 2026 Sep 23;18(5):plag043. doi: 10.1093/aobpla/plag043 (PMC13643487; doi:10.1093/aobpla/plag043)
Supplement: Supplementary file 1 [file plag043_supplementary_data.zip › SuppFigs_merged_compressed.pdf]

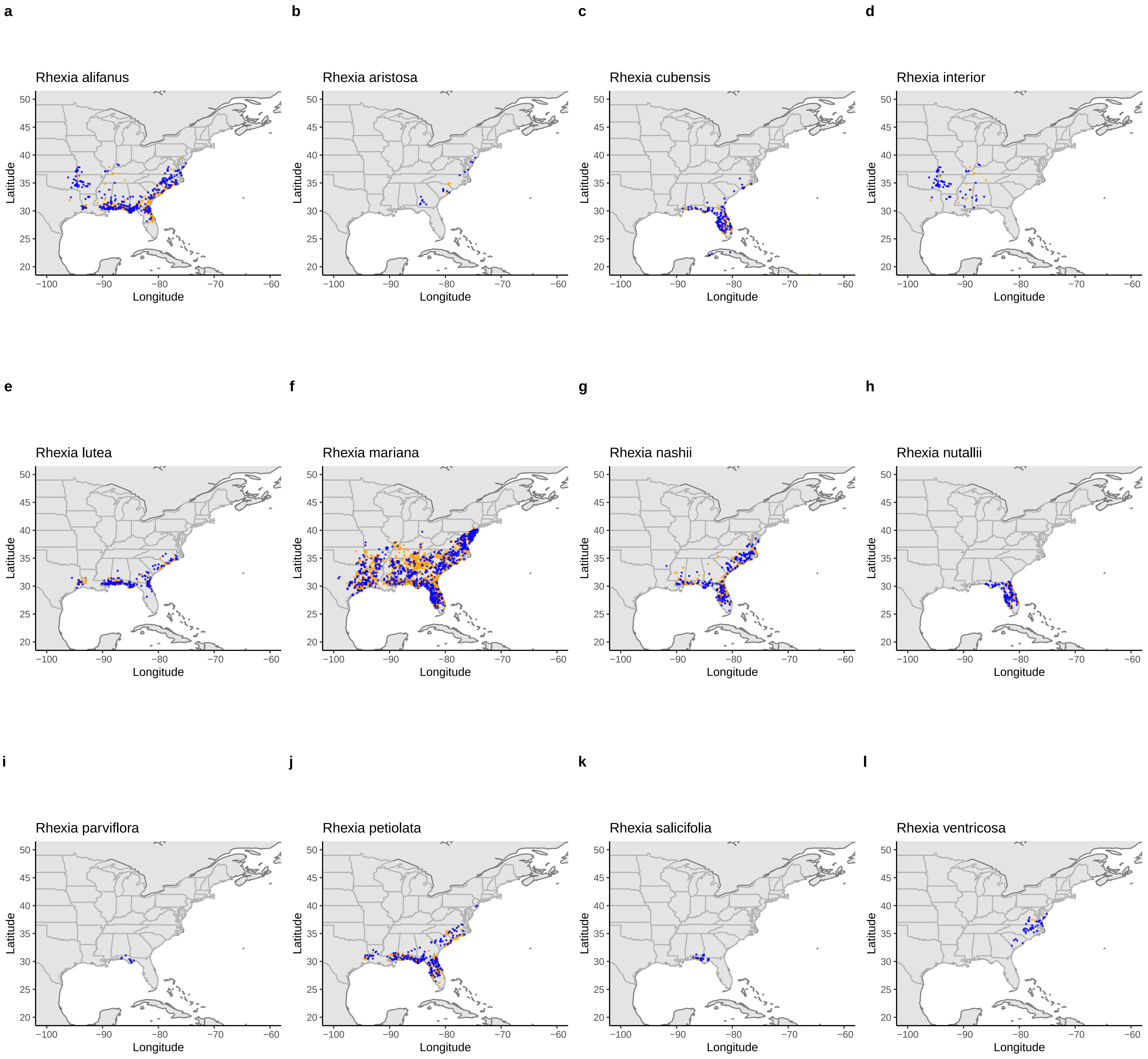

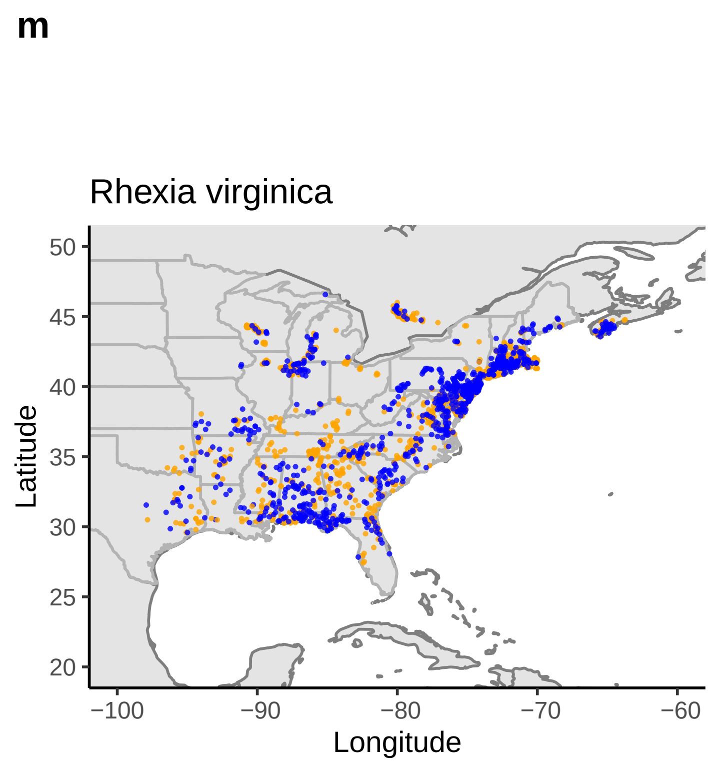

Supplementary Figure 1. Cleaned data maps for each species, color-coded by Basis of Record: blue represents preserved specimens, while orange indicates human observations. A) *R. alifanus*, B) *R. aristosa*, C) *R. cubensis*, D) *R. interior*, E) *R. lutea*, F) *R. mariana*, G) *R. nashii*, H) *R. nuttallii*, I) *R. parviflora*, J) *R. petiolata*, K) *R. salicifolia*, L) *R. ventricosa*, and M) *R. virginica*.

Basis of Record · HUMAN_OBSERVATION · PRESERVED_SPECIMEN

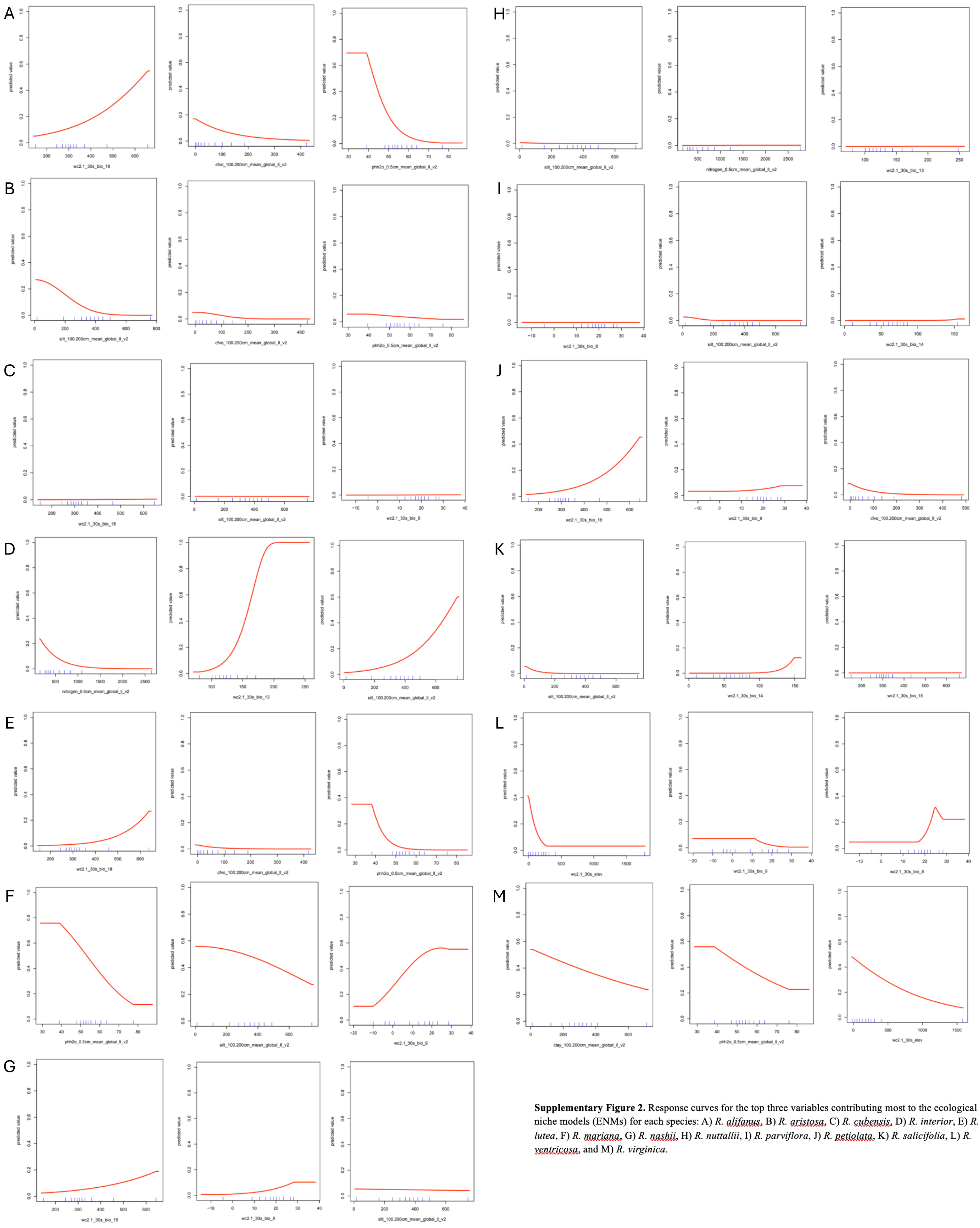

Supplementary Figure 2. Response curves for the top three variables contributing most to the ecological niche models (ENMs) for each species: A) *R. alifanus*, B) *R. aristosa*, C) *R. cubensis*, D) *R. interior*, E) *R. lutea*, F) *R. mariana*, G) *R. nashii*, H) *R. nuttallii*, I) *R. parviflora*, J) *R. petiolata*, K) *R. salicifolia*, L) *R. ventricosa*, and M) *R. virginica*.

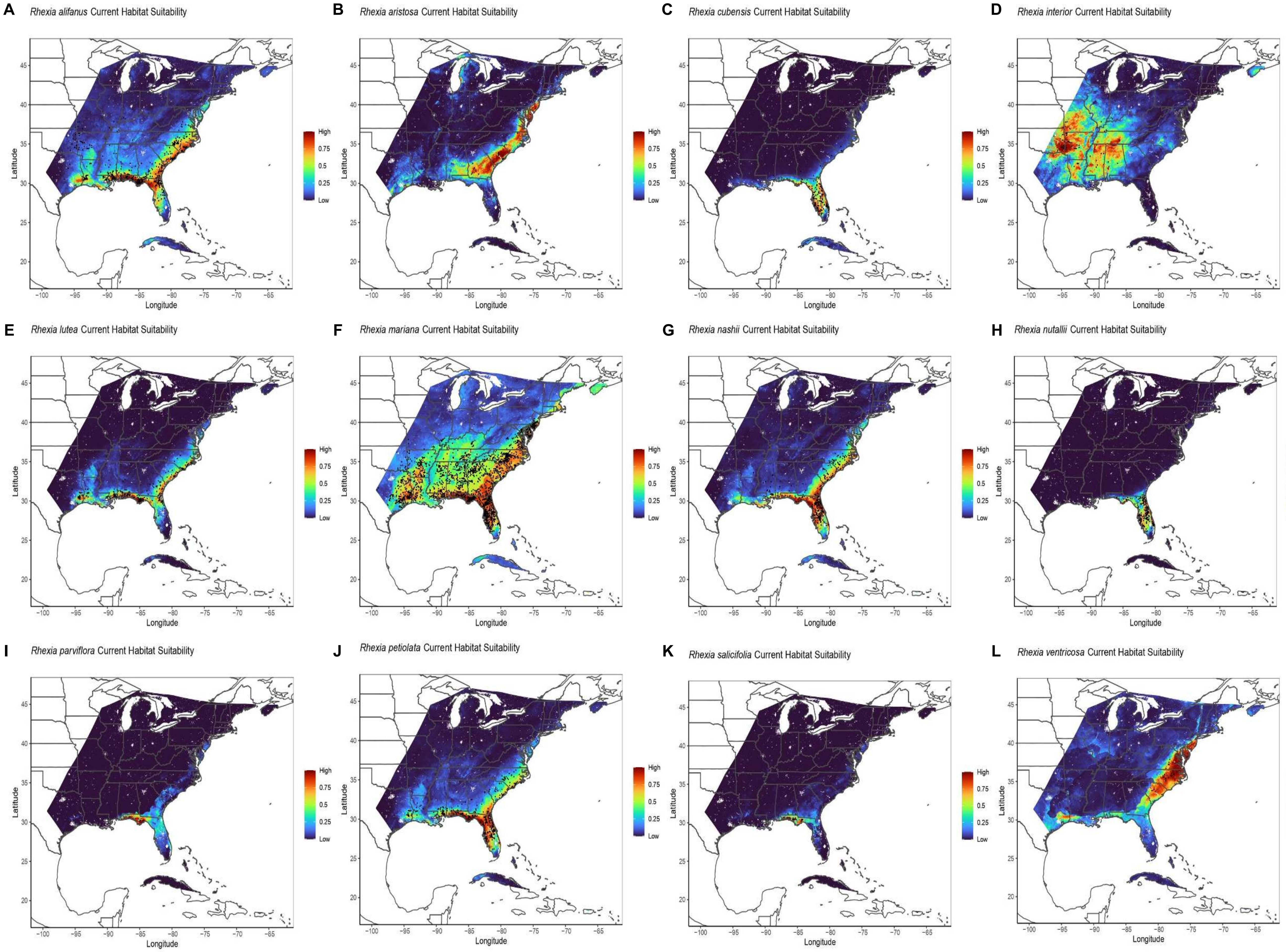

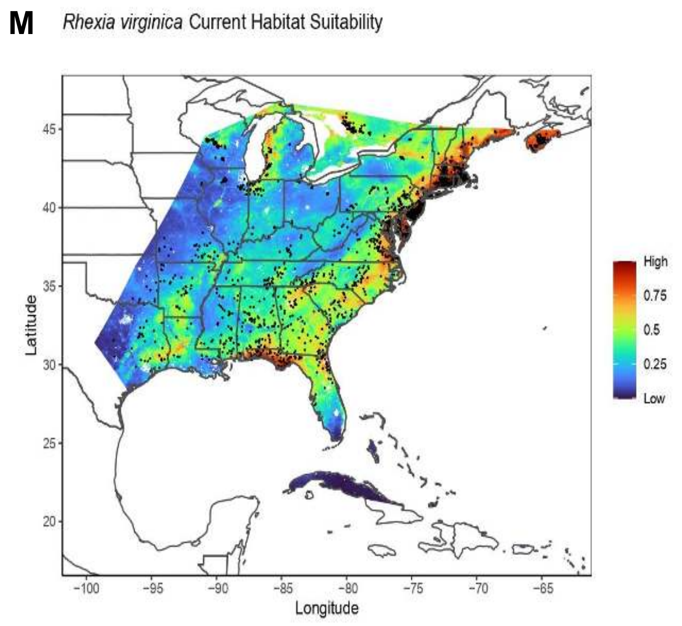

Supplementary Figure 3. Current ENM projections. A) *R. alifanus*, B) *R. aristosa*, C) *R. cubensis*, D) *R. interior*, E) *R. lutea*, F) *R. mariana*, G) *R. nashii*, H) *R. nuttallii*, I) *R. parviflora*, J) *R. petiolata*, K) *R. salicifolia*, L) *R. ventricosa*, and M) *R. virginica*.

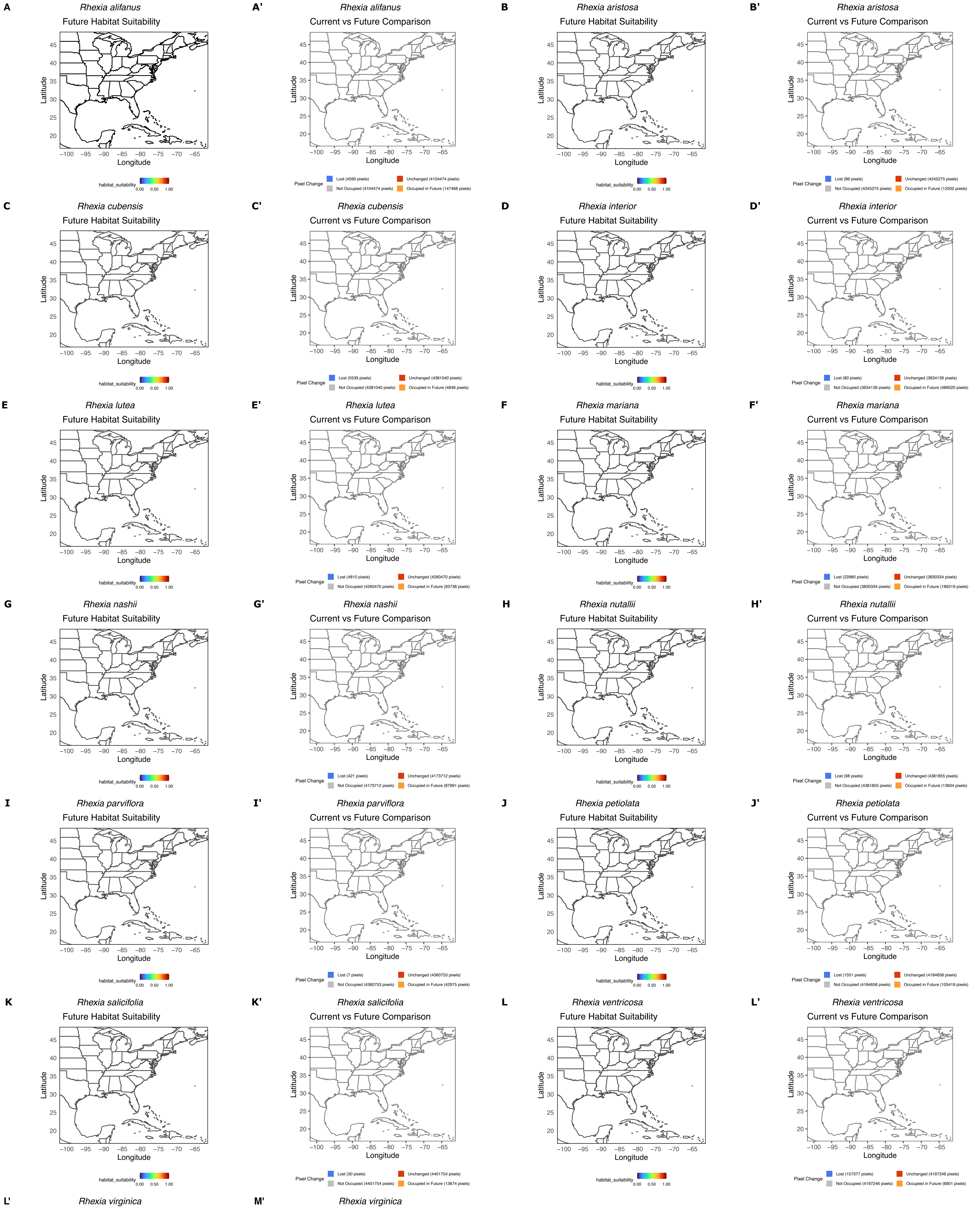

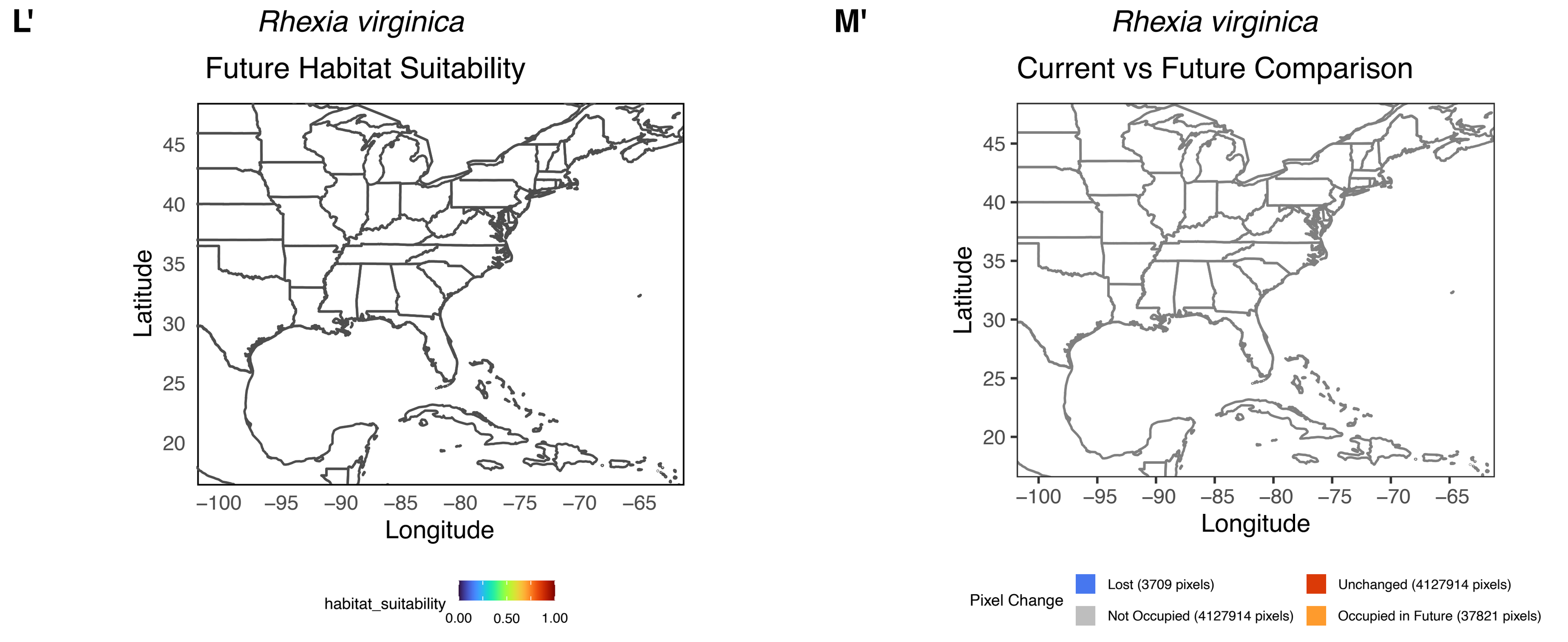

Supplementary Figure 4. Future habitat suitability projections and distribution changes for *Rhexia* species under the ACCESS-CM2 climate model for the 2041–2060 period under the SSP2-4.5 scenario. Panels (A–M) show future habitat suitability projections, with suitability values ranging from 0 (unsuitable) to 1 (highly suitable). Panels (A'–M') present comparisons between current and future habitat suitability, categorizing areas as lost (red), unchanged (gray), not occupied (white), or newly occupied in the future (blue). These projections highlight potential habitat shifts under future climate conditions.

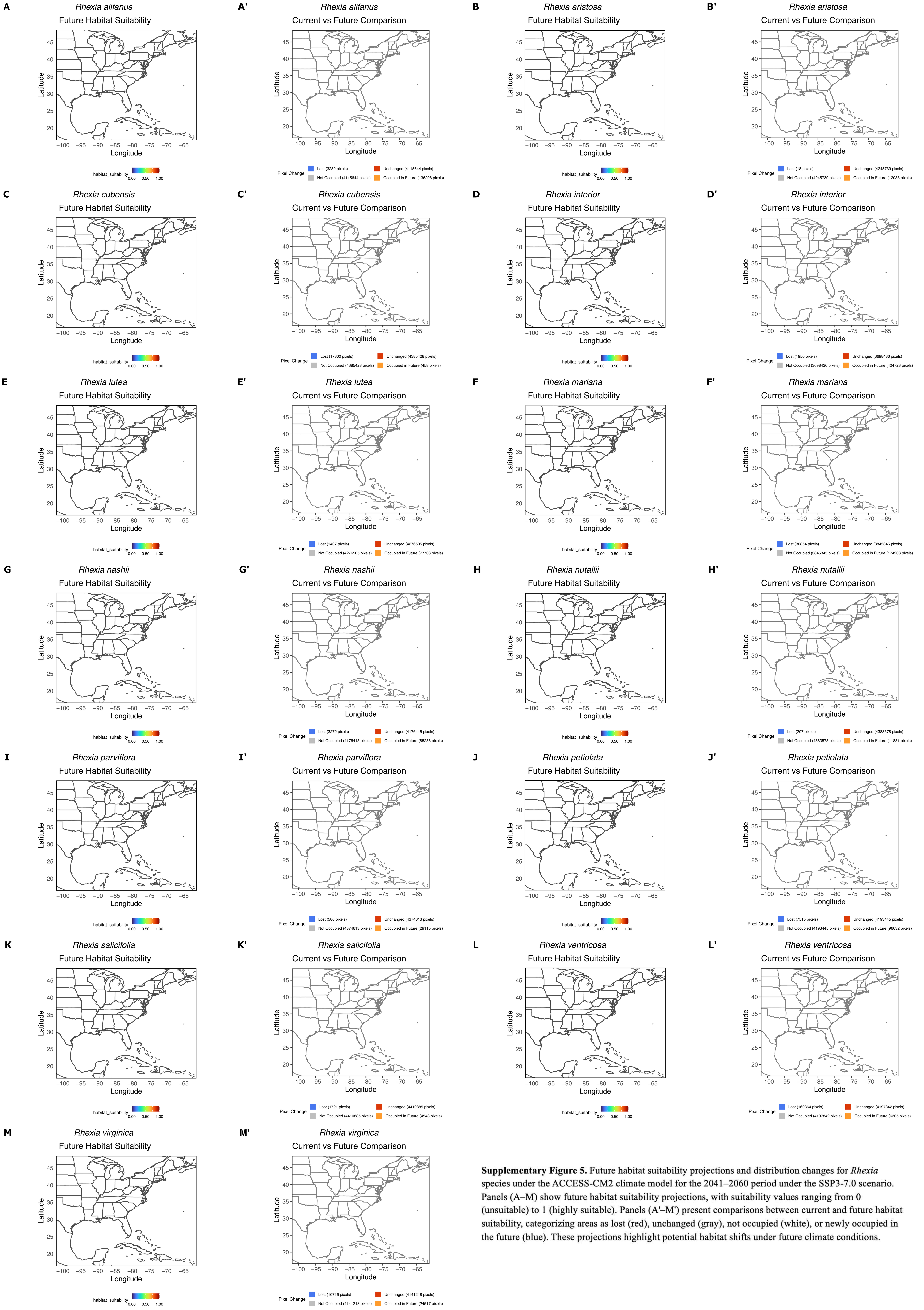

Supplementary Figure 5. Future habitat suitability projections and distribution changes for *Rhexia* species under the ACCESS-CM2 climate model for the 2041–2060 period under the SSP3-7.0 scenario. Panels (A–M) show future habitat suitability projections, with suitability values ranging from 0 (unsuitable) to 1 (highly suitable). Panels (A'–M') present comparisons between current and future habitat suitability, categorizing areas as lost (red), unchanged (gray), not occupied (white), or newly occupied in the future (blue). These projections highlight potential habitat shifts under future climate conditions.

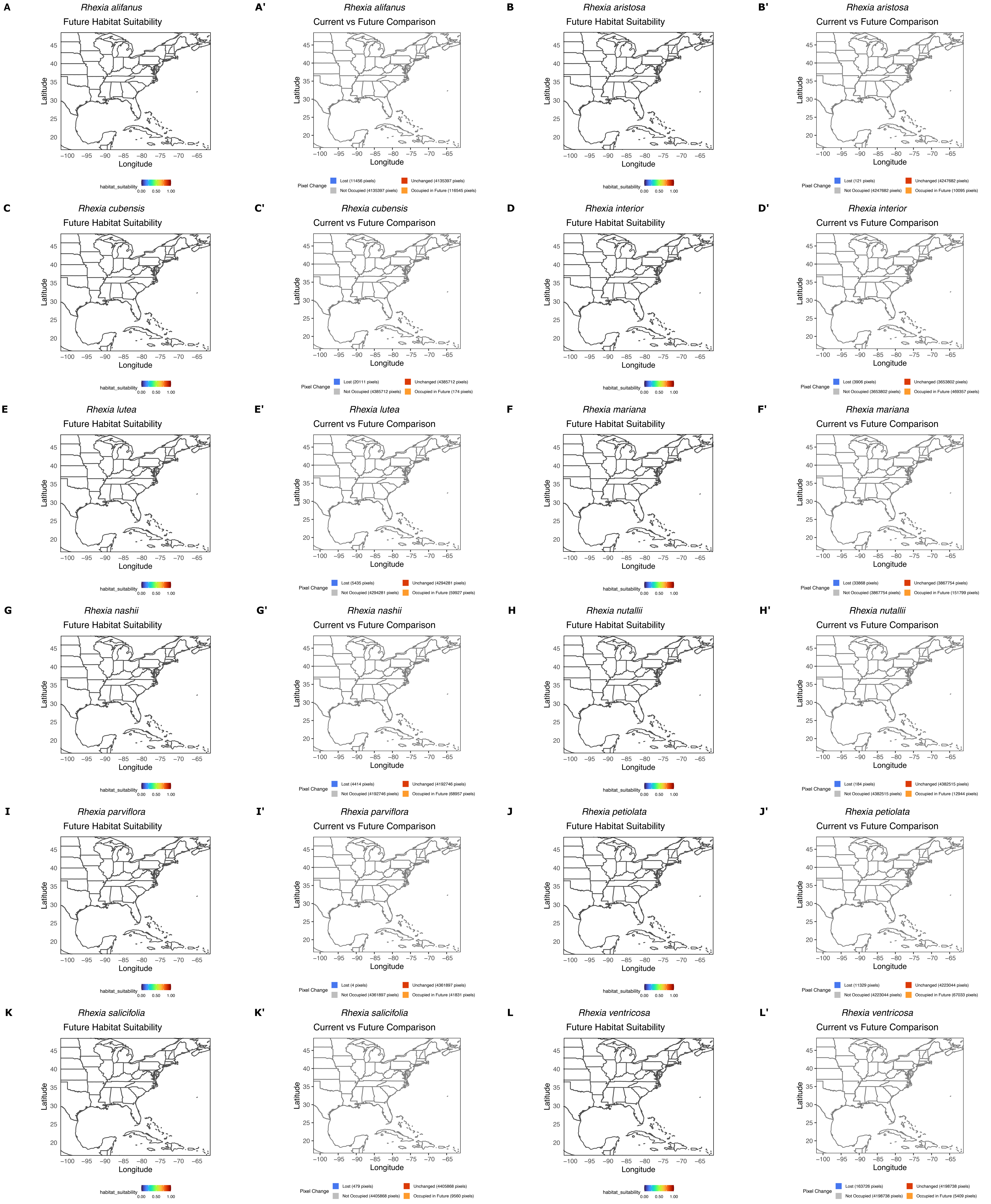

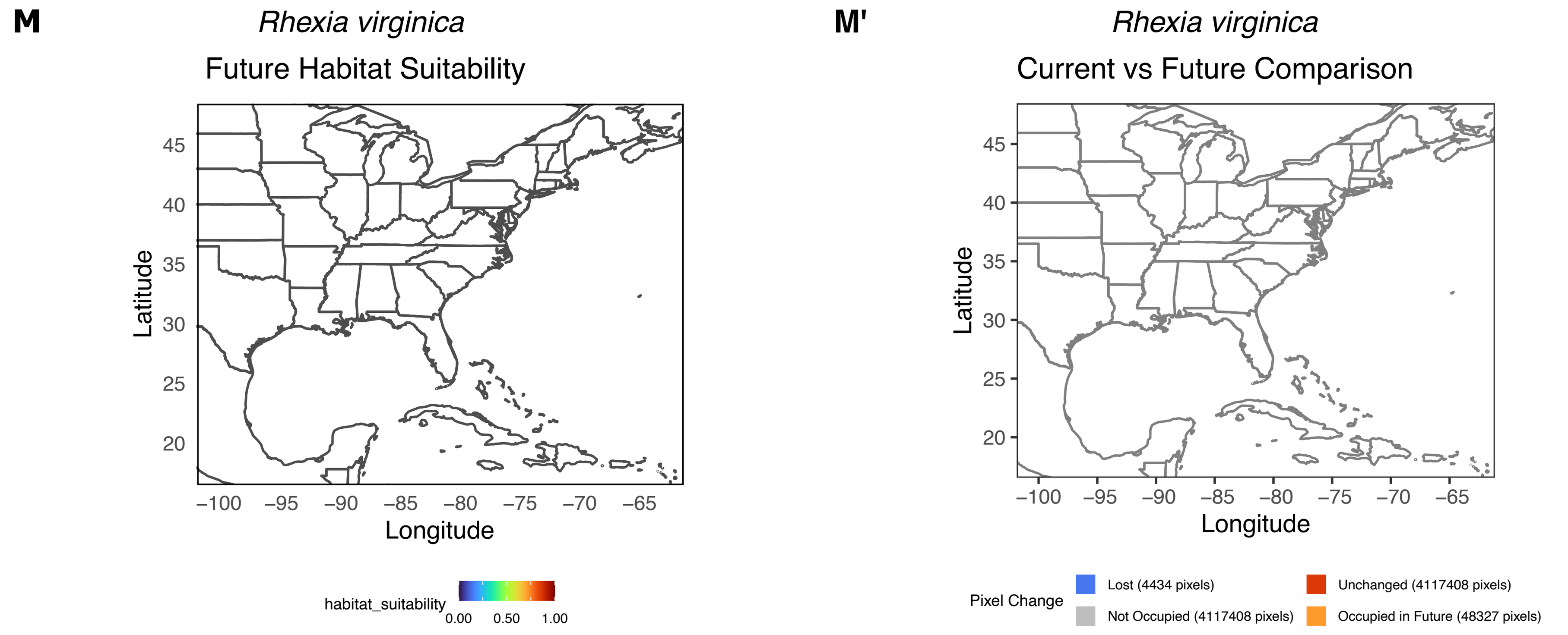

Supplementary Figure 6. Future habitat suitability projections and distribution changes for *Rhexia* species under the ACCESS-CM2 climate model for the 2041–2060 period under the SSP5-8.5 scenario. Panels (A–M) show future habitat suitability projections, with suitability values ranging from 0 (unsuitable) to 1 (highly suitable). Panels (A'–M') present comparisons between current and future habitat suitability, categorizing areas as lost (red), unchanged (gray), not occupied (white), or newly occupied in the future (blue). These projections highlight potential habitat shifts under future climate conditions.

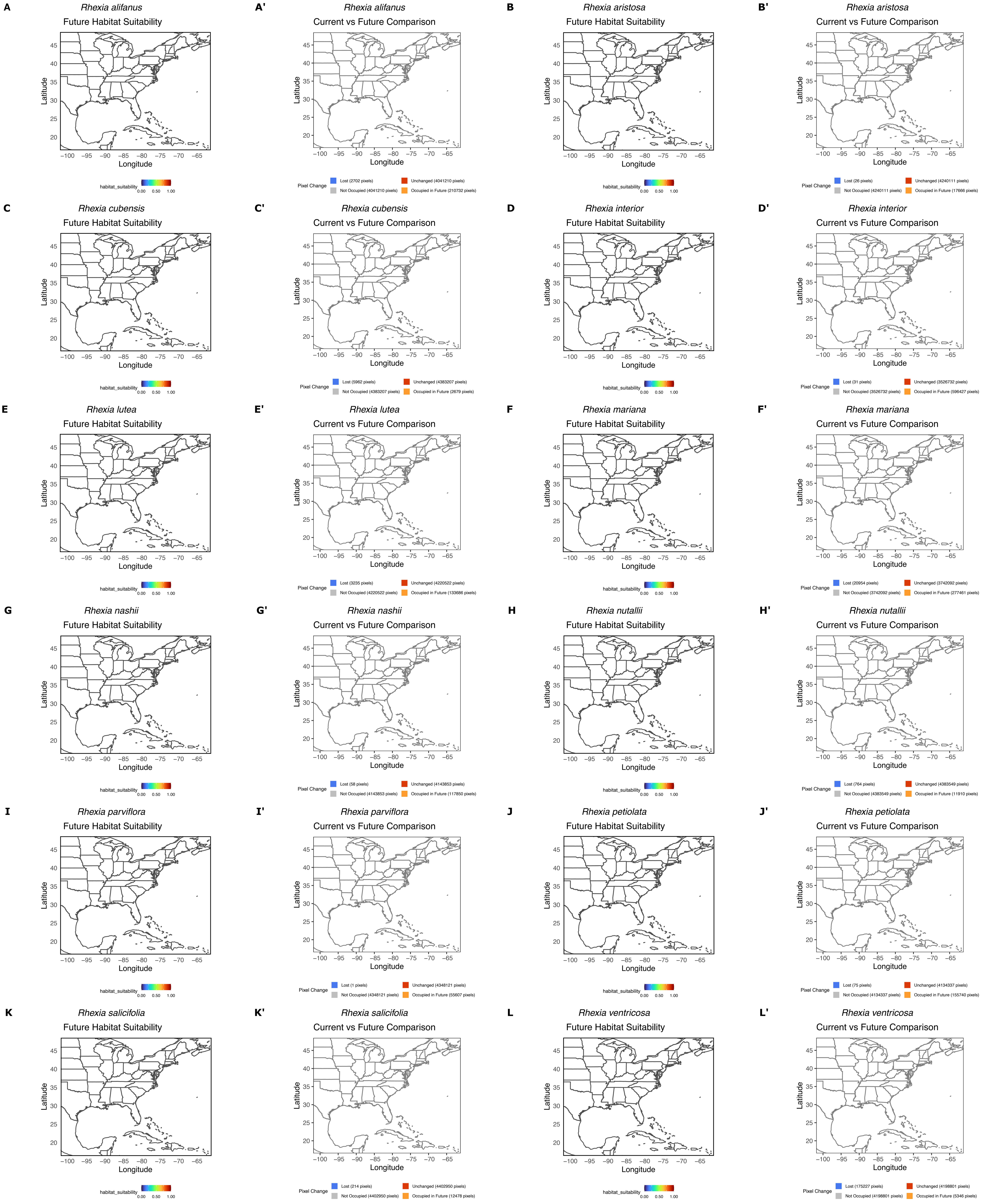

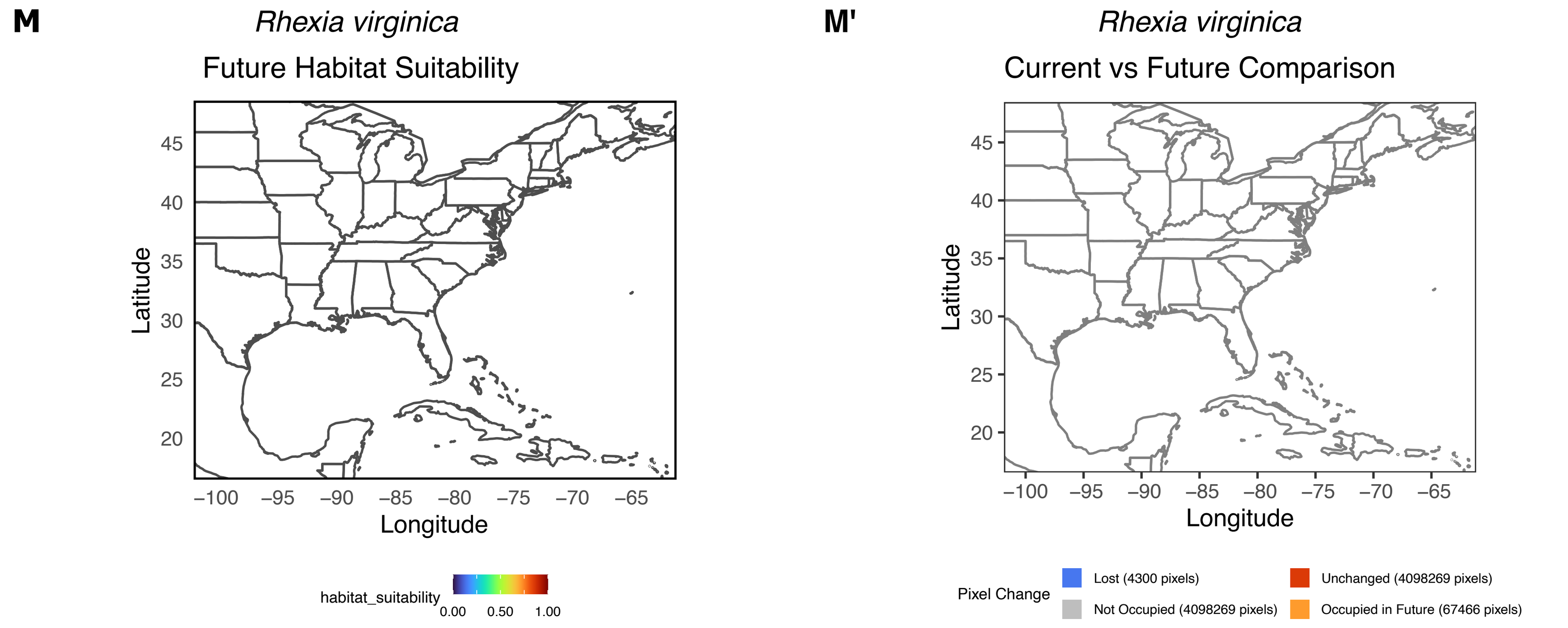

Supplementary Figure 7. Future habitat suitability projections and distribution changes for *Rhexia* species under the ACCESS-CM2 climate model for the 2081–2100 period under the SSP2-4.5 scenario. Panels (A–M) show future habitat suitability projections, with suitability values ranging from 0 (unsuitable) to 1 (highly suitable). Panels (A'–M') present comparisons between current and future habitat suitability, categorizing areas as lost (red), unchanged (gray), not occupied (white), or newly occupied in the future (blue). These projections highlight potential habitat shifts under future climate conditions.

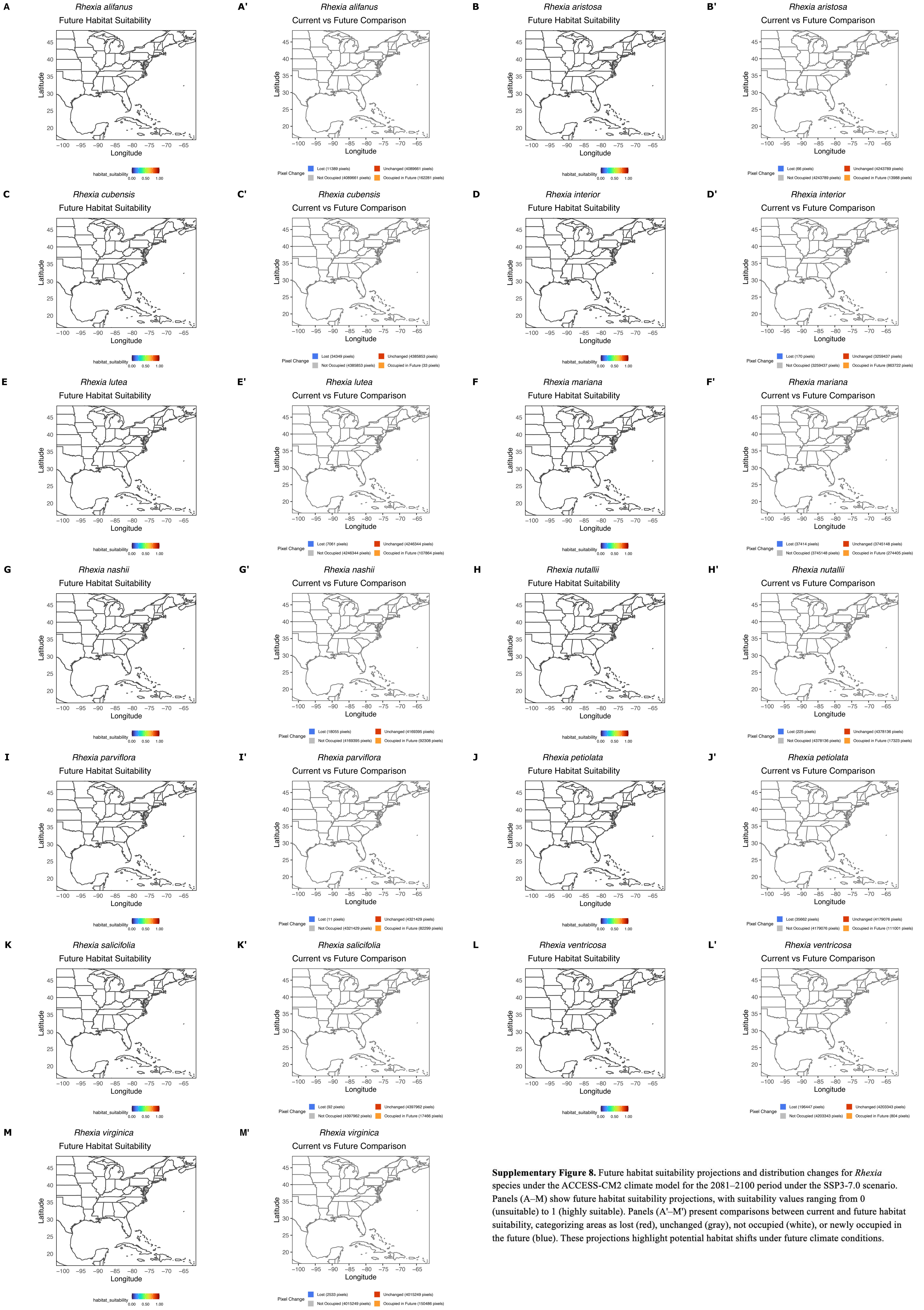

Supplementary Figure 8. Future habitat suitability projections and distribution changes for *Rhexia* species under the ACCESS-CM2 climate model for the 2081–2100 period under the SSP3-7.0 scenario. Panels (A–M) show future habitat suitability projections, with suitability values ranging from 0 (unsuitable) to 1 (highly suitable). Panels (A'–M') present comparisons between current and future habitat suitability, categorizing areas as lost (red), unchanged (gray), not occupied (white), or newly occupied in the future (blue). These projections highlight potential habitat shifts under future climate conditions.

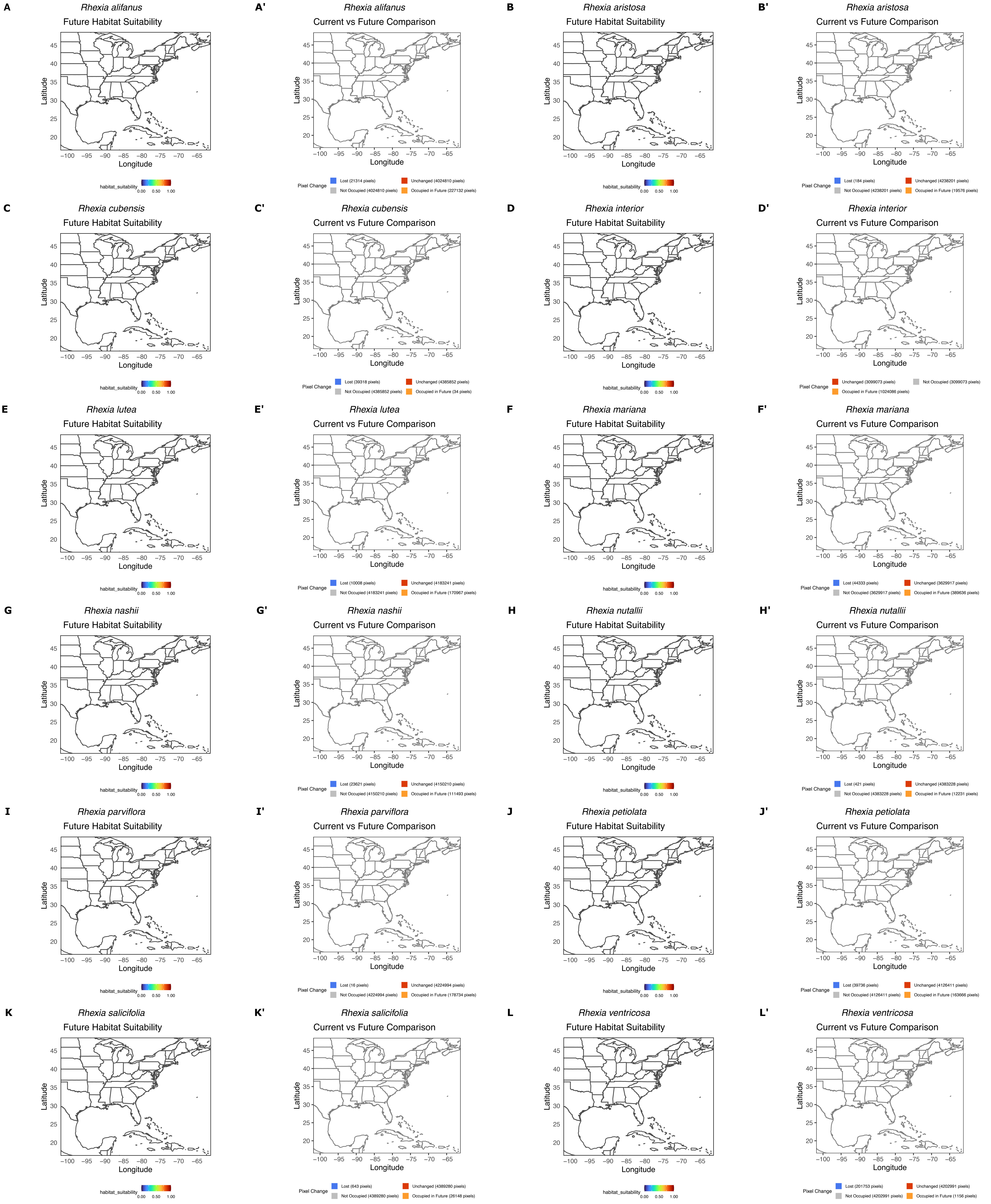

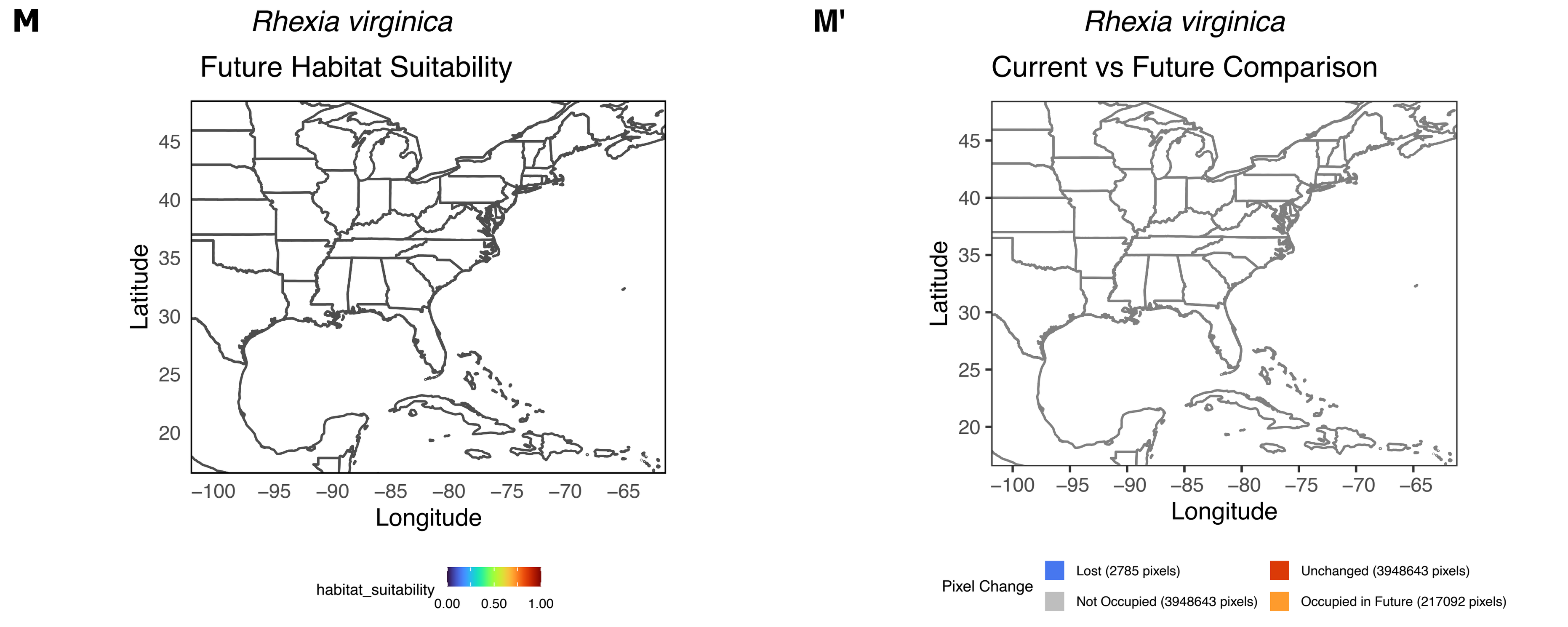

Supplementary Figure 9. Future habitat suitability projections and distribution changes for *Rhexia* species under the ACCESS-CM2 climate model for the 2081–2100 period under the SSP5-8.5 scenario. Panels (A–M) show future habitat suitability projections, with suitability values ranging from 0 (unsuitable) to 1 (highly suitable). Panels (A'–M') present comparisons between current and future habitat suitability, categorizing areas as lost (red), unchanged (gray), not occupied (white), or newly occupied in the future (blue). These projections highlight potential habitat shifts under future climate conditions.

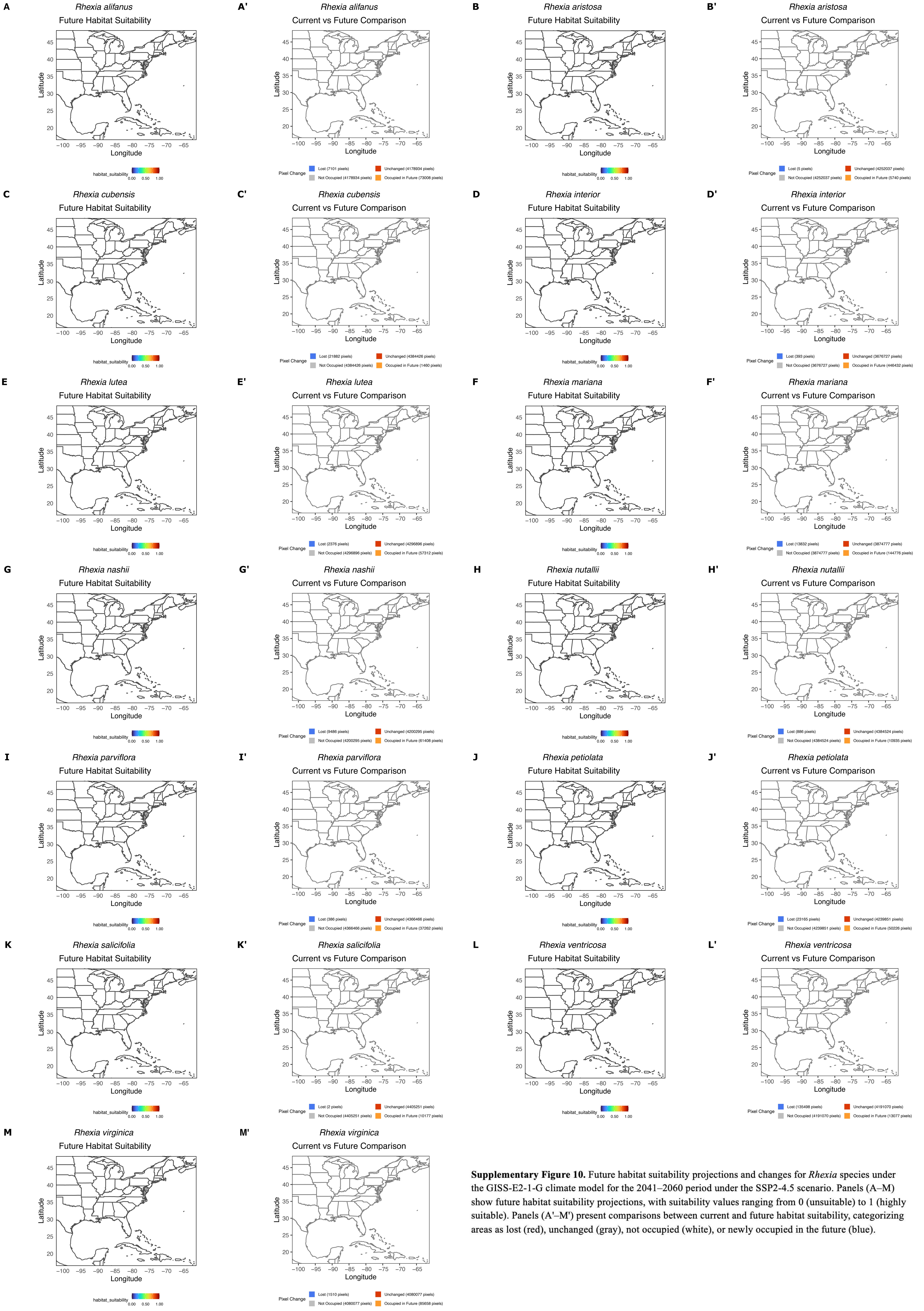

Supplementary Figure 10. Future habitat suitability projections and changes for *Rhexia* species under the GISS-E2-1-G climate model for the 2041–2060 period under the SSP2-4.5 scenario. Panels (A–M) show future habitat suitability projections, with suitability values ranging from 0 (unsuitable) to 1 (highly suitable). Panels (A'–M') present comparisons between current and future habitat suitability, categorizing areas as lost (red), unchanged (gray), not occupied (white), or newly occupied in the future (blue).

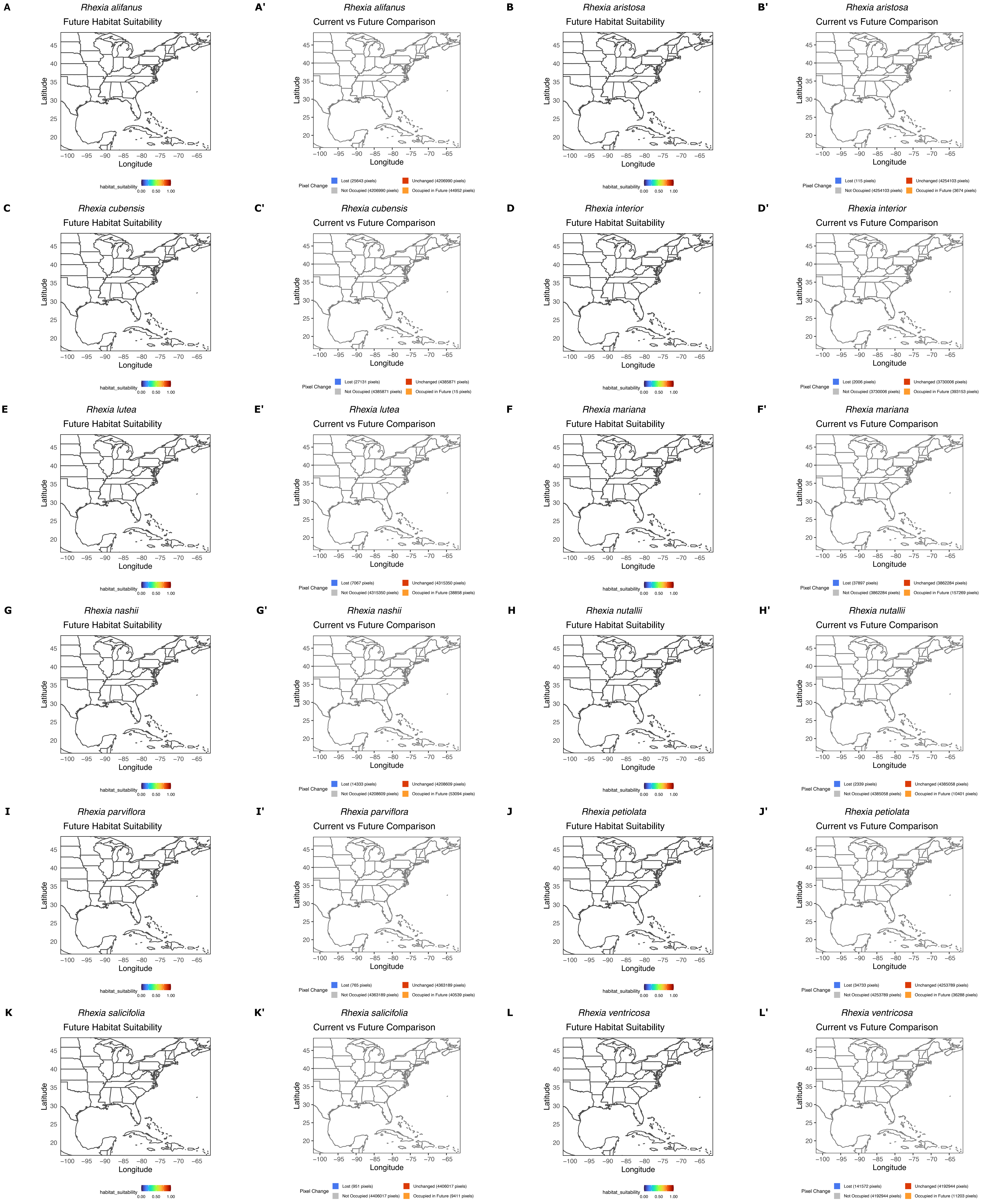

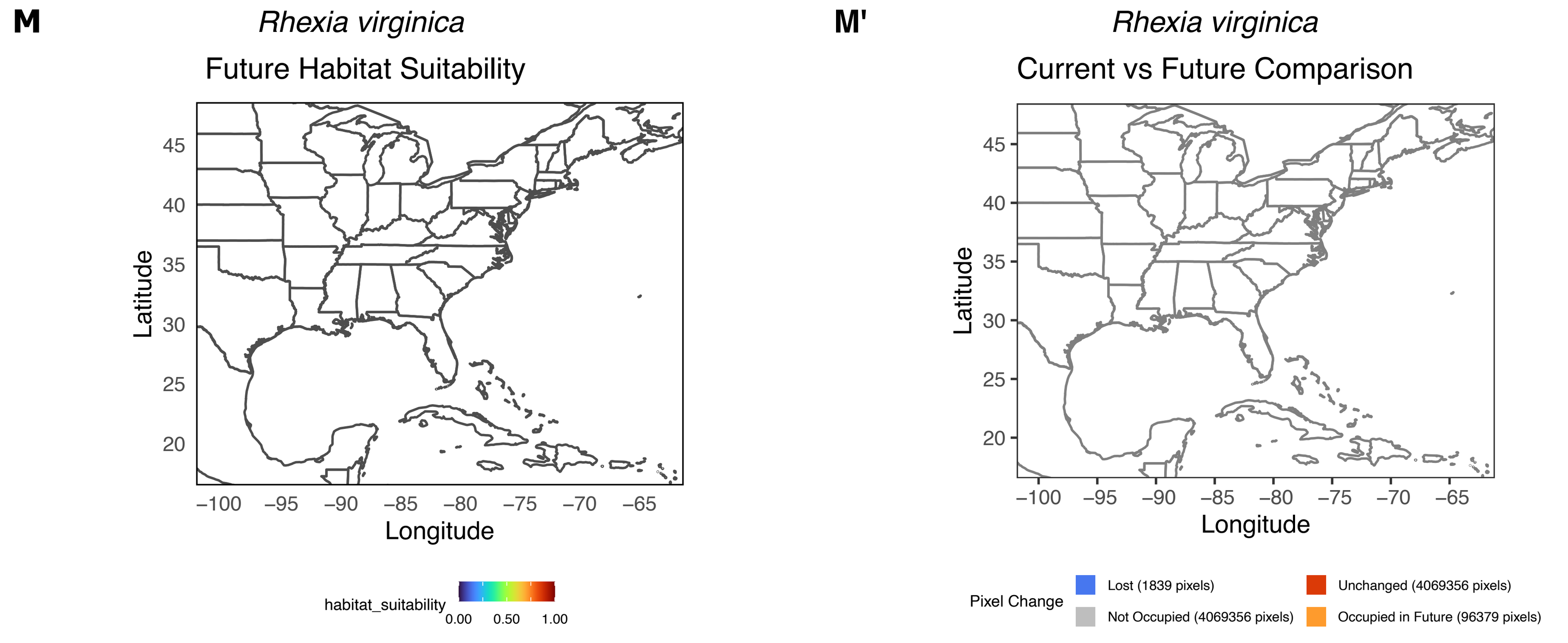

Supplementary Figure 11. Future habitat suitability projections and changes for *Rhexia* species under the GISS-E2-1-G climate model for the 2041–2060 period under the SSP3-7.0 scenario. Panels (A–M) show future habitat suitability projections, with suitability values ranging from 0 (unsuitable) to 1 (highly suitable). Panels (A'–M') present comparisons between current and future habitat suitability, categorizing areas as lost (red), unchanged (gray), not occupied (white), or newly occupied in the future (blue).

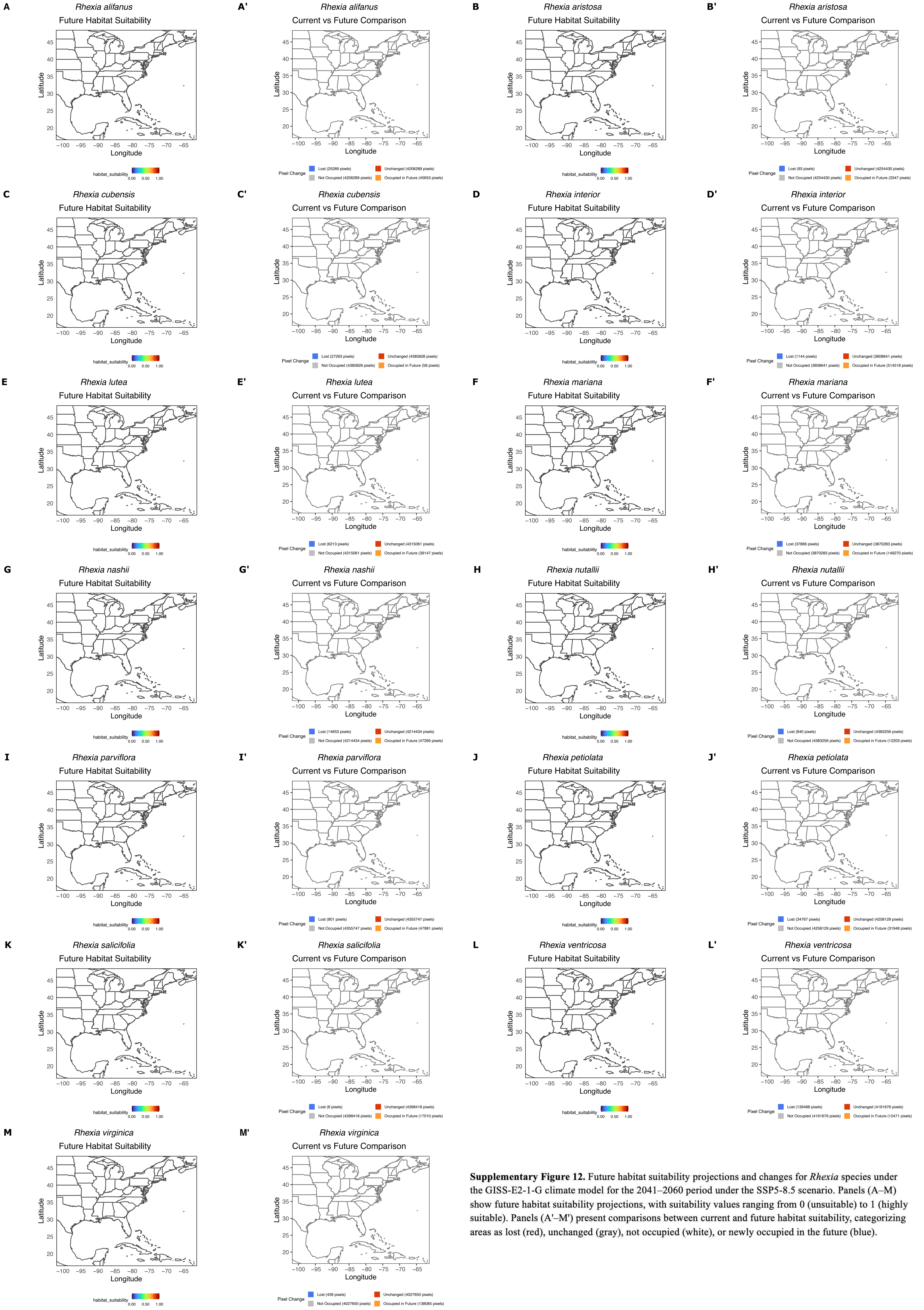

Supplementary Figure 12. Future habitat suitability projections and changes for *Rhexia* species under the GISS-E2-1-G climate model for the 2041–2060 period under the SSP5-8.5 scenario. Panels (A–M) show future habitat suitability projections, with suitability values ranging from 0 (unsuitable) to 1 (highly suitable). Panels (A'–M') present comparisons between current and future habitat suitability, categorizing areas as lost (red), unchanged (gray), not occupied (white), or newly occupied in the future (blue).

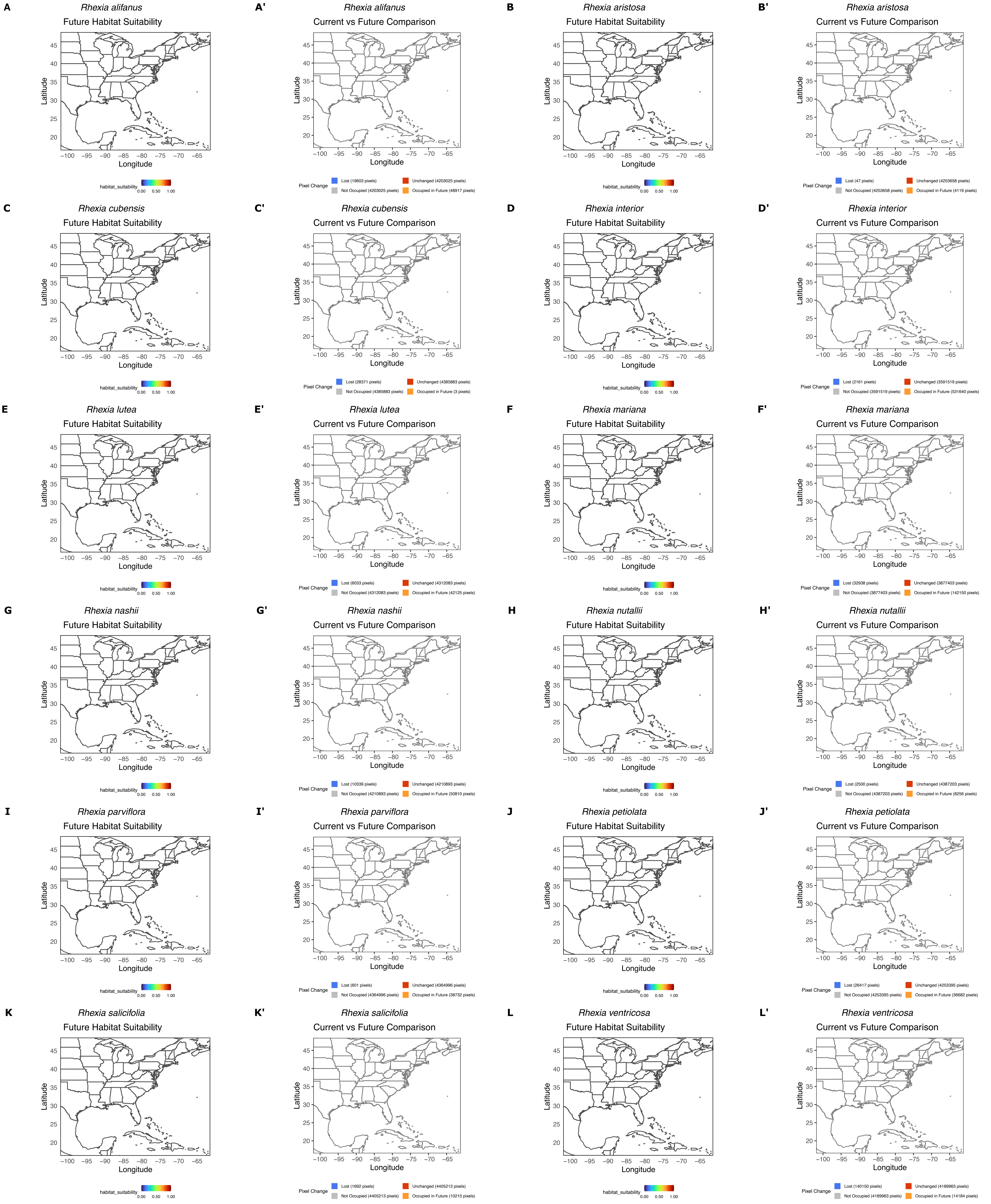

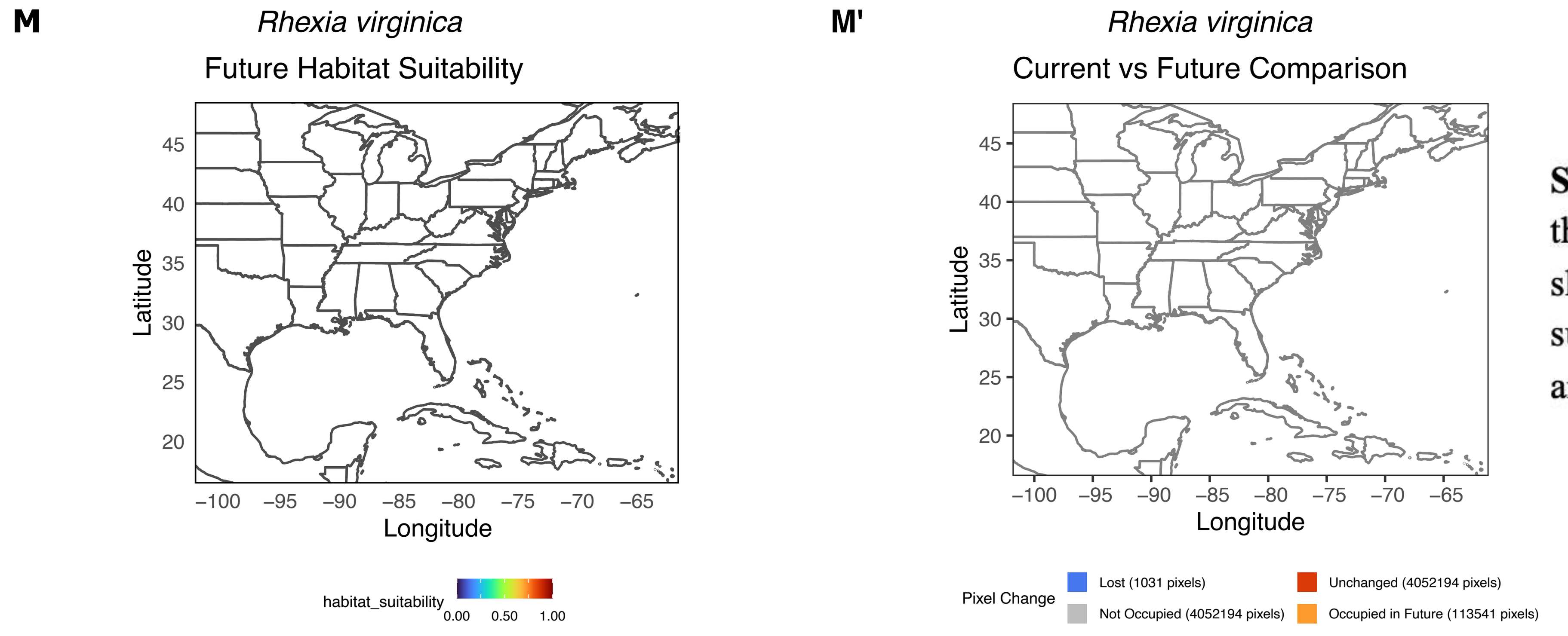

Supplementary Figure 13. Future habitat suitability projections and changes for *Rhexia* species under the GISS-E2-1-G climate model for the 2081–2100 period under the SSP2-4.5 scenario. Panels (A–M) show future habitat suitability projections, with suitability values ranging from 0 (unsuitable) to 1 (highly suitable). Panels (A'–M') present comparisons between current and future habitat suitability, categorizing areas as lost (red), unchanged (gray), not occupied (white), or newly occupied in the future (blue).

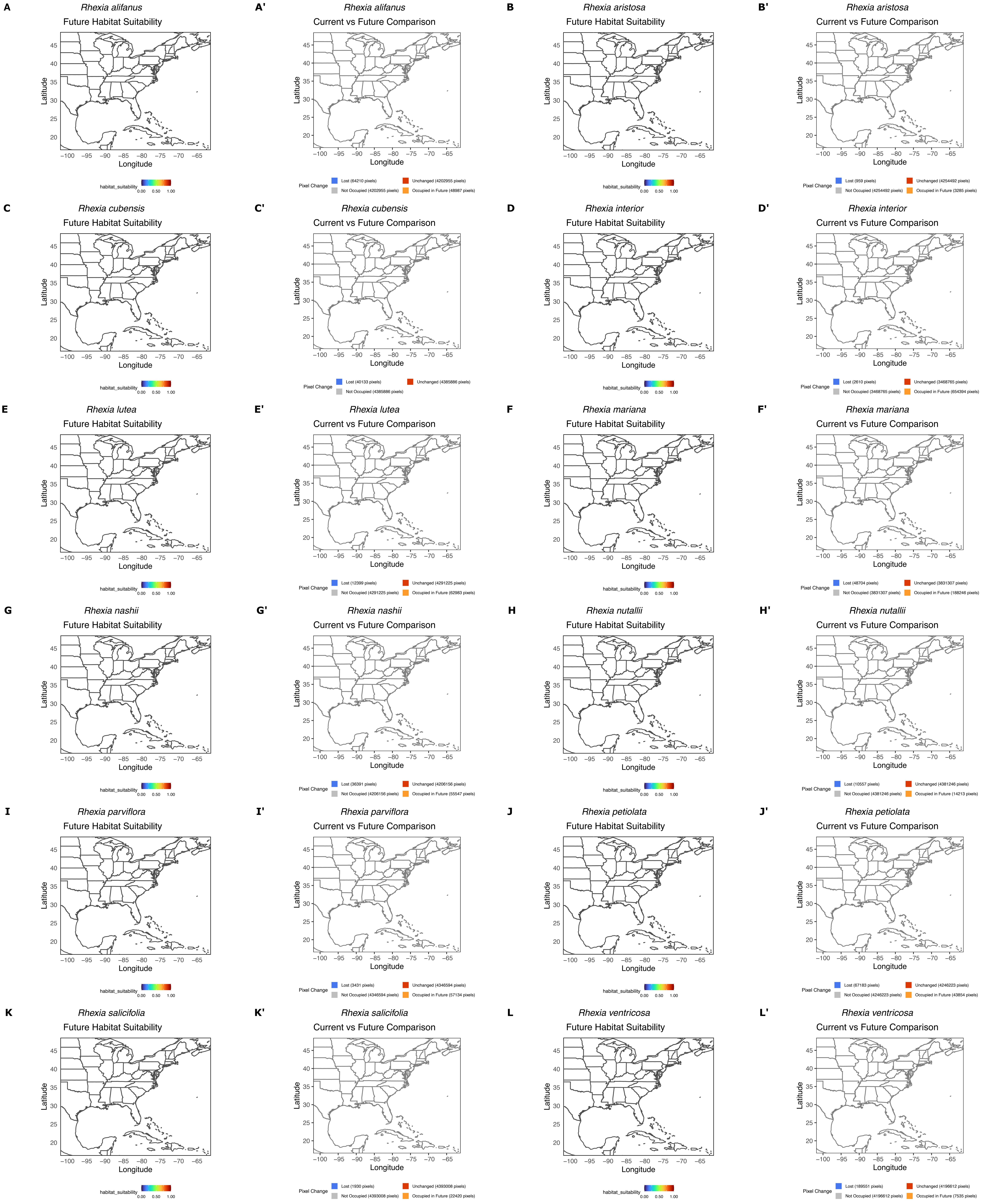

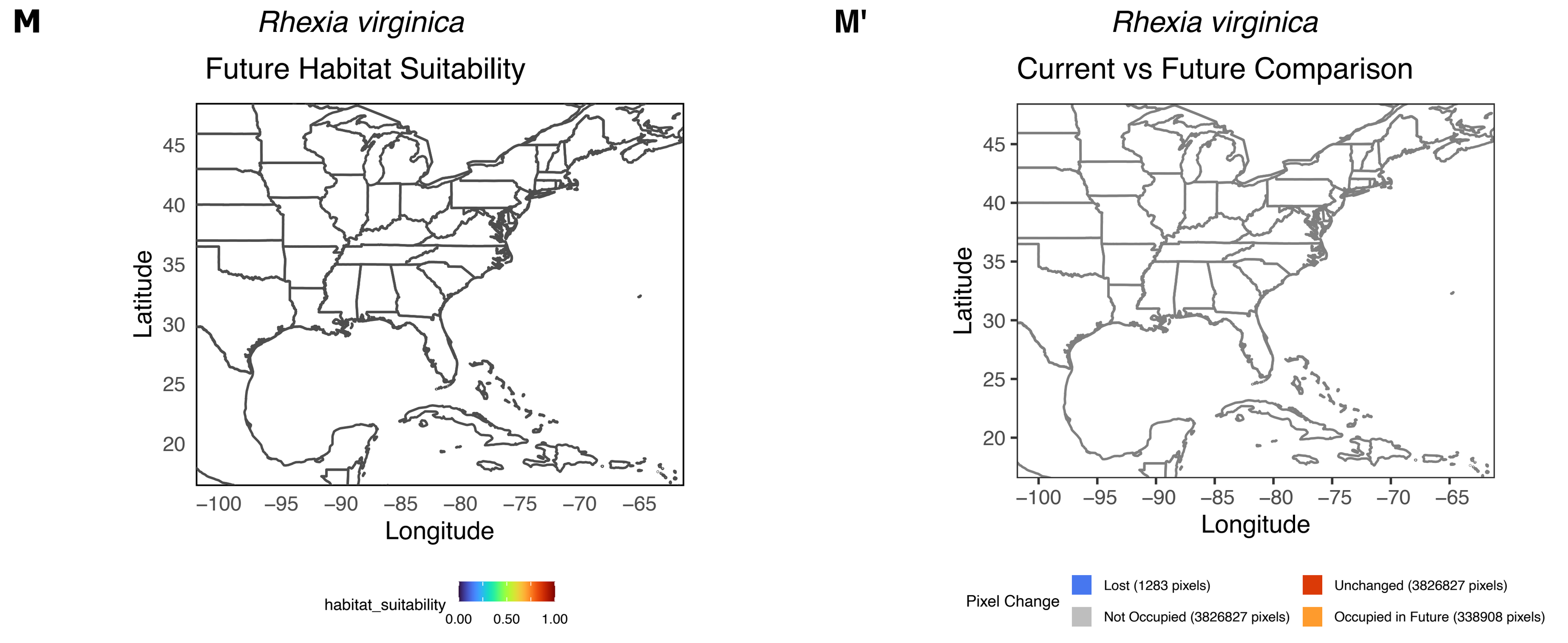

Supplementary Figure 14. Future habitat suitability projections and changes for *Rhexia* species under the GISS-E2-1-G climate model for the 2081–2100 period under the SSP3-7.0 scenario. Panels (A–M) show future habitat suitability projections, with suitability values ranging from 0 (unsuitable) to 1 (highly suitable). Panels (A'–M') present comparisons between current and future habitat suitability, categorizing areas as lost (red), unchanged (gray), not occupied (white), or newly occupied in the future (blue).

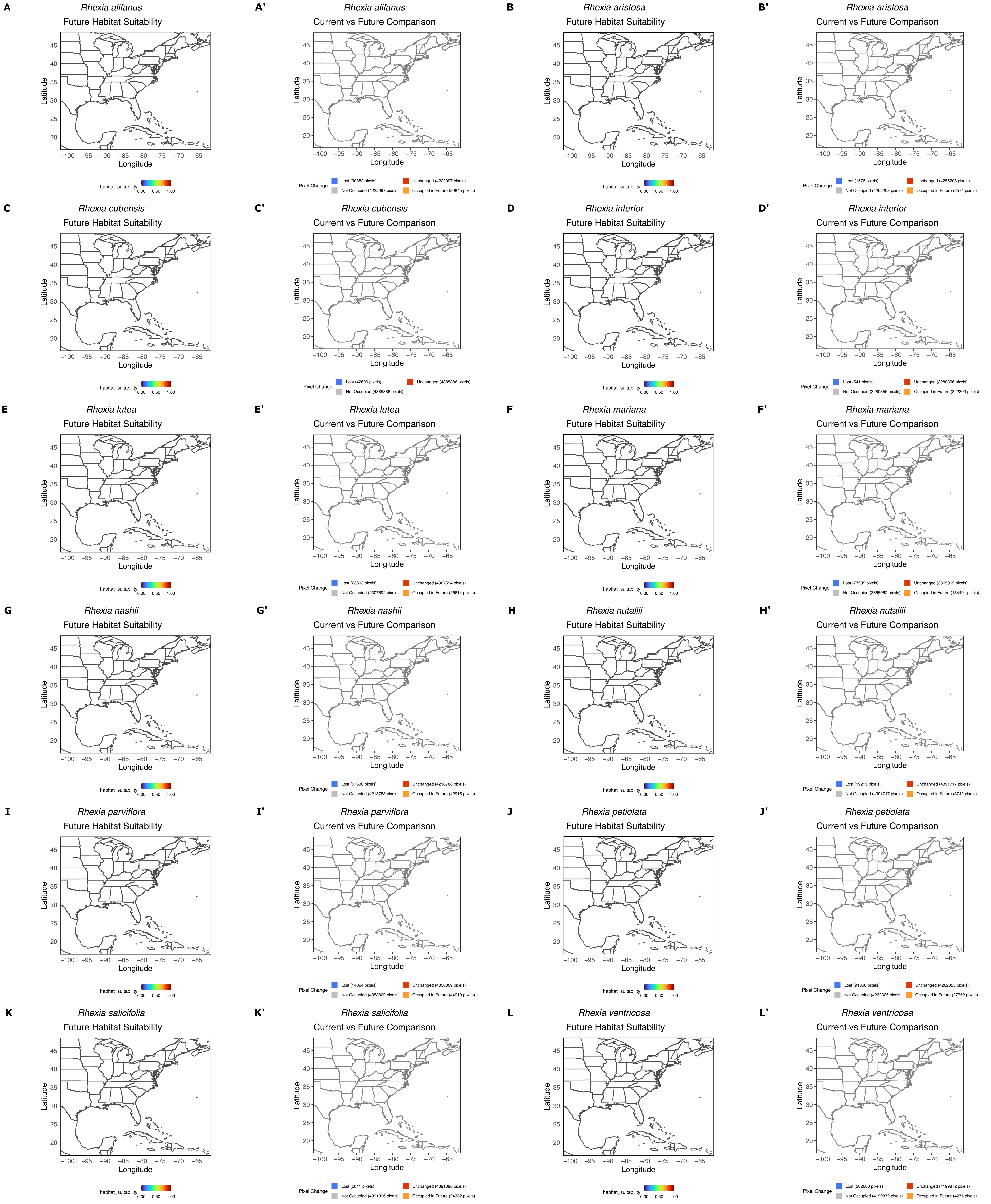

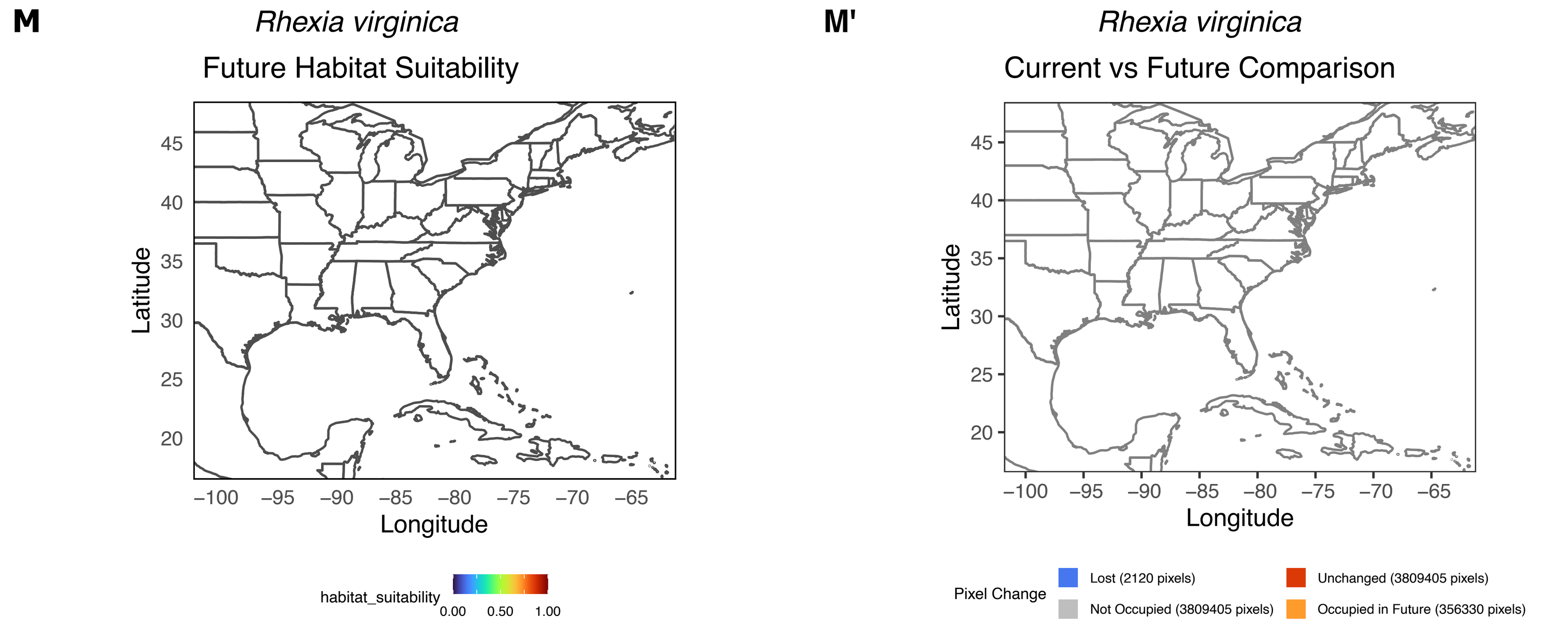

Supplementary Figure 15. Future habitat suitability projections and changes for *Rhexia* species under the GISS-E2-1-G climate model for the 2081–2100 period under the SSP5-8.5 scenario. Panels (A–M) show future habitat suitability projections, with suitability values ranging from 0 (unsuitable) to 1 (highly suitable). Panels (A'–M') present comparisons between current and future habitat suitability, categorizing areas as lost (red), unchanged (gray), not occupied (white), or newly occupied in the future (blue).

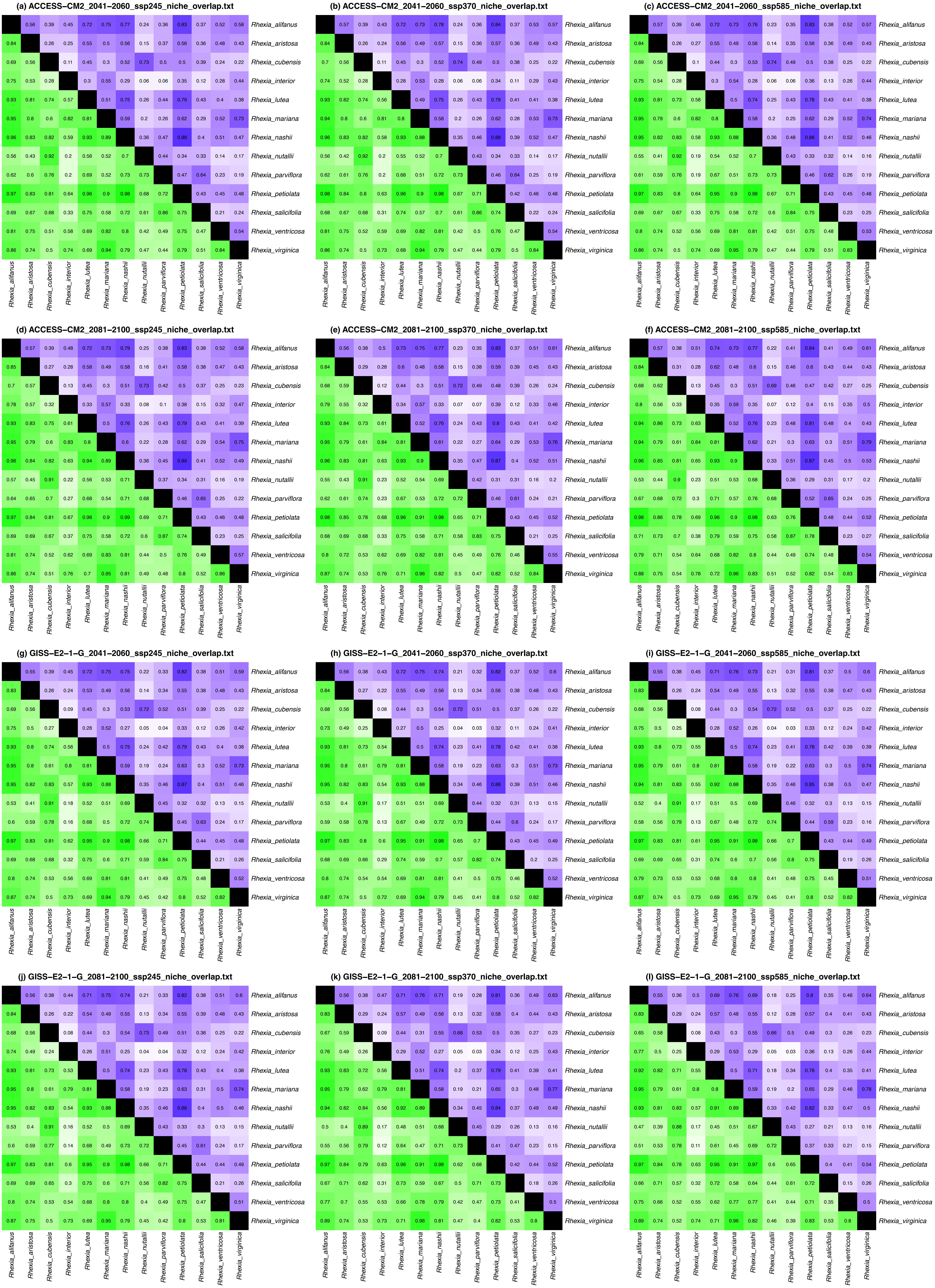

Supplementary Figure 16. Niche overlap matrices for *Rhexia* species under future climate projections. Each panel represents niche overlap estimates for different climate models (ACCESS-CM2, GISS-E2-1-G) and time periods (2041–2060, 2081–2100) under SSP 2.6, 3.7, and 5.8 scenarios. Schoener's D values (upper triangle, blue) indicate absolute niche overlap, while Warren's I values (lower triangle, green) incorporate probability-weighted similarity.
