# Supplementary material for: Forecasts of climate-driven habitat suitability distribution are complicated by taxonomic and cytogenetic complexity in Rhexia (Melastomataceae)
Source: AoB Plants. 2026 Sep 23;18(5):plag043. doi: 10.1093/aobpla/plag043 (PMC13643487; doi:10.1093/aobpla/plag043)
Supplement: Supplementary file 1 [file plag043_supplementary_data.zip › SuppTable1_Taxonomy_DataCounts.docx]

**Supplementary Table 1.** Occurrence data was downloaded from GBIF and iDigBio for each species (Raw Occurrence Records) and filtered to remove outliers and duplicates (Cleaned Occurrence Records).

| **Species** | **Series** | **Chromosome Count** | **Sections: Newsom 2012** | **Groups: Newsom 2012** | **Raw Data** | **Cleaned Data** |
| --- | --- | --- | --- | --- | --- | --- |
| *Rhexia alifanus* | B | n = 11 | CYMBORHEXIA | - | 1662 | 807 |
| *Rhexia aristosa* | B | n = 11 | RHEXIA | Equales | 37 | 25 |
| *Rhexia cubensis* | B | n = 11, 22, 33 | RHEXIA | Inaequales | 566 | 247 |
| *Rhexia interior* | - | n = 22 | RHEXIA | Equales | 216 | 79 |
| *Rhexia lutea* | A | n = 22 | LUTEORHEXIA | - | 928 | 361 |
| *Rhexia mariana* | B | n = 11 | RHEXIA | Inaequales | 5714 | 2930 |
| *Rhexia nashii* | B | n = 22, 33 | RHEXIA | Inaequales | 1009 | 433 |
| *Rhexia nuttallii* | A | n = 11 | BREVIANTHERA | - | 570 | 235 |
| *Rhexia parviflora* | A or B | n = 11 | RHEXIA | Inaequales | 39 | 9 |
| *Rhexia petiolata* | A | n = 11 | BREVIANTHERA | - | 1154 | 487 |
| *Rhexia salicifolia* | B | n = 11 | RHEXIA | Equales | 134 | 28 |
| *Rhexia ventricosa* | - | n = 22 | RHEXIA | Equales | 115 | 42 |
| *Rhexia virginica* | B | n = 11, 22 | RHEXIA | Equales | 4255 | 1971 |
