# Supplementary material for: Forecasts of climate-driven habitat suitability distribution are complicated by taxonomic and cytogenetic complexity in Rhexia (Melastomataceae)
Source: AoB Plants. 2026 Sep 23;18(5):plag043. doi: 10.1093/aobpla/plag043 (PMC13643487; doi:10.1093/aobpla/plag043)
Supplement: Supplementary file 1 [file plag043_supplementary_data.zip › SuppTable2_ENMresults_V2.docx]

**Supplementary Table 2:** Optimal model and top three variables with percent contributed and correlated variables.

| Species | Optimal Model | Top Variables (%) | Top Correlated  Variables | Second Variables (%) | Second Correlated  Variables | Third Variables (%) | Third Correlated  Variables |
| --- | --- | --- | --- | --- | --- | --- | --- |
| *Rhexia alifanus* | fc.L_rm.4.5 | Bio_18 (41.5%) | Bio_13,  Bio_16 | cfvo_100.200cm (24.1%) | cfvo_0.5cm,  cfvo_5.15cm,  cfvo_15.30cm,  cfvo_30.60cm,  cfvo_60.100cm,  elev | phh2o_0.5cm (22.9%) | phh2o_5.15cm,  phh2o_15.30cm,  phh2o_30.60cm,  phh2o_60.100cm,  phh2o_100.200cm, |
| *Rhexia aristosa* | fc.LQ_rm.2.5 | Silt_100.200cm (39.6%) | Sand_0.5cm,  Sand_5.15cm,  Sand_15.30cm,  Sand_30.60cm,  Sand_60.100cm,  Sand_100.200cm,  Silt_0.5cm,  Silt_5.15cm,  Silt_15.30cm,  Silt_30.60cm,  Silt_60.100cm | cfvo_100.200cm (23.9%) | cfvo_0.5cm,  cfvo_5.15cm,  cfvo_15.30cm,  cfvo_30.60cm,  cfvo_60.100cm,  elev | phh2o_0.5cm (10.0%) | phh2o_5.15cm,  phh2o_15.30cm,  phh2o_30.60cm,  phh2o_60.100cm,  phh2o_100.200cm, |
| *Rhexia cubensis* | fc.L_rm.0.5 | Bio_18 (41.0%) | Bio_13,  Bio_16 | silt_100.200cm (30.8%) | Sand_0.5cm,  Sand_5.15cm,  Sand_15.30cm,  Sand_30.60cm,  Sand_60.100cm, Sand_100.200cm,  Silt_0.5cm,  Silt_5.15cm,  Silt_15.30cm,  Silt_30.60cm,  Silt_60.100cm | Bio_8 (8.8%) | **NONE** |
| *Rhexia interior* | fc.L_rm.4.5 | Nitrogen_0.5cm (41.0%) | Nitrogen_5.15cm,  Nitrogen_15.30cm | Bio_13 (17.8%) | Bio_1,  Bio_3,  Bio_4,  Bio_6,  Bio_7,  Bio_11,  Bio_12,  Bio_16,  Bio_18 | silt_100.200cm (11.4%) | Sand_0.5cm,  Sand_5.15cm,  Sand_15.30cm,  Sand_30.60cm,  Sand_60.100cm,  Sand_100.200cm,  Silt_0.5cm,  Silt_5.15cm,  Silt_15.30cm,  Silt_30.60cm,  Silt_60.100cm |
| *Rhexia lutea* | fc.L_rm.2.5 | Bio_18 (35.9%) | Bio_13,  Bio_16 | cfvo_100.200cm (28.0%) | cfvo_0.5cm,  cfvo_5.15cm,  cfvo_15.30cm,  cfvo_30.60cm,  cfvo_60.100cm,  elev | phh2o_0.5cm (17.6%) | phh2o_5.15cm,  phh2o_15.30cm,  phh2o_30.60cm,  phh2o_60.100cm,  phh2o_100.200cm, |
| *Rhexia mariana* | fc.LQ_rm.4.5 | Bio_9 (26.7%) | Bio_1, Bio_3,  Bio_4,  Bio_5,  Bio_6,  Bio_10,  Bio_11,  Bio_12 | silt_100.200cm (18.4%) | Sand_0.5cm,  Sand_5.15cm,  Sand_15.30cm,  Sand_30.60cm,  Sand_60.100cm, Sand_100.200cm,  Silt_0.5cm,  Silt_5.15cm,  Silt_15.30cm,  Silt_30.60cm,  Silt_60.100cm | phh2o_0.5cm (14.7%) | phh2o_5.15cm,  phh2o_15.30cm,  phh2o_30.60cm,  phh2o_60.100cm,  phh2o_100.200cm |
| *Rhexia nashii* | fc.L_rm.4.5 | Bio_18 (37.1%) | Bio_13,  Bio_16 | Bio_8 (17.3%) | **NONE** | silt_100.200cm (16.5%) | Sand_0.5cm,  Sand_5.15cm,  Sand_15.30cm,  Sand_30.60cm,  Sand_60.100cm,  Sand_100.200cm,  Silt_0.5cm,  Silt_5.15cm,  Silt_15.30cm,  Silt_30.60cm,  Silt_60.100cm |
| *Rhexia nuttallii* | fc.H_rm.3.5 | Silt_100.200cm (67.5%) | Sand_0.5cm,  Sand_5.15cm,  Sand_15.30cm,  Sand_30.60cm,  Sand_60.100cm, Sand_100.200cm,  Silt_0.5cm,  Silt_5.15cm,  Silt_15.30cm,  Silt_30.60cm,  Silt_60.100cm | nitrogen_0.5cm (21.1%) | Nitrogen_5.15cm, Nitrogen_15.30cm | Bio_13 (8.3%) | Bio_1,  Bio_3,  Bio_4,  Bio_6,  Bio_7,  Bio_11,  Bio_12,  Bio_16,  Bio_18 |
| *Rhexia parviflora* | fc.LQ_rm.4.5 | Bio_8 (40.9%) | **NONE** | silt_100.200cm (38.0%) | Sand_0.5cm,  Sand_5.15cm,  Sand_15.30cm,  Sand_30.60cm,  Sand_60.100cm, Sand_100.200cm,  Silt_0.5cm,  Silt_5.15cm,  Silt_15.30cm,  Silt_30.60cm,  Silt_60.100cm | Bio_14 (20.6%) | Bio_12,  Bio_17,  Bio_19 |
| *Rhexia petiolata* | fc.LQ_rm.4.5 | Bio_18 (31.5%) | Bio_13,  Bio_16 | Bio_8 (25.0%) | **NONE** | cfvo_100.200cm (13.9%) | cfvo_0.5cm,  cfvo_5.15cm,  cfvo_15.30cm,  cfvo_30.60cm,  cfvo_60.100cm,  elev |
| *Rhexia salicifolia* | fc.L_rm.3.5 | Silt_100.200cm (40.3%) | Sand_0.5cm,  Sand_5.15cm,  Sand_15.30cm,  Sand_30.60cm,  Sand_60.100cm, Sand_100.200cm,  Silt_0.5cm,  Silt_5.15cm,  Silt_15.30cm,  Silt_30.60cm,  Silt_60.100cm | Bio_14 (18.9%) | Bio_12,  Bio_17,  Bio_19 | Bio_18 (17.2%) | Bio_13, Bio_16 |
| *Rhexia ventricosa* | fc.H_rm.4.5 | Elev (38.2%) | cfvo_100.200cm | Bio_9 (22.2%) | Bio_1,  Bio_3,  Bio_4,  Bio_5,  Bio_6,  Bio_10,  Bio_11,  Bio_12 | Bio_8 (16.6%) | **NONE** |
| *Rhexia virginica* | fc.L_rm.4.5 | Clay_100.200cm (35.1%) | Clay_15.30cm,  Clay_30.60cm,  Clay_60.100cm,  Sand_30.60cm,  Sand_60.100cm,  Sand_100.200cm | phh2o_0.5cm (31.9%) | phh2o_5.15cm,  phh2o_15.30cm,  phh2o_30.60cm,  phh2o_60.100cm,  phh2o_100.200cm, | Elev (10.2%) | cfvo_100.200cm |
